# Large-Scale Machine Learning Analysis Reveals DNA Methylation and Gene Expression Response Signatures for Gemcitabine-Treated Pancreatic Cancer

**DOI:** 10.34133/hds.0108

**Published:** 2024-01-08

**Authors:** Adeolu Ogunleye, Chayanit Piyawajanusorn, Ghita Ghislat, Pedro J. Ballester

**Affiliations:** ^1^ Department of Organismal Biology, Uppsala University, Uppsala, Sweden.; ^2^ Department of Bioengineering, Imperial College London, London, UK.; ^3^ Department of Life Sciences, Imperial College London, London, UK.

## Abstract

**Background:** Gemcitabine is a first-line chemotherapy for pancreatic adenocarcinoma (PAAD), but many PAAD patients do not respond to gemcitabine-containing treatments. Being able to predict such nonresponders would hence permit the undelayed administration of more promising treatments while sparing gemcitabine life-threatening side effects for those patients. Unfortunately, the few predictors of PAAD patient response to this drug are weak, none of them exploiting yet the power of machine learning (ML). **Methods:** Here, we applied ML to predict the response of PAAD patients to gemcitabine from the molecular profiles of their tumors. More concretely, we collected diverse molecular profiles of PAAD patient tumors along with the corresponding clinical data (gemcitabine responses and clinical features) from the Genomic Data Commons resource. From systematically combining 8 tumor profiles with 16 classification algorithms, each of the resulting 128 ML models was evaluated by multiple 10-fold cross-validations. **Results:** Only 7 of these 128 models were predictive, which underlines the importance of carrying out such a large-scale analysis to avoid missing the most predictive models. These were here random forest using 4 selected mRNAs [0.44 Matthews correlation coefficient (MCC), 0.785 receiver operating characteristic–area under the curve (ROC-AUC)] and XGBoost combining 12 DNA methylation probes (0.32 MCC, 0.697 ROC-AUC). By contrast, the hENT1 marker obtained much worse random-level performance (practically 0 MCC, 0.5 ROC-AUC). Despite not being trained to predict prognosis (overall and progression-free survival), these ML models were also able to anticipate this patient outcome. **Conclusions:** We release these promising ML models so that they can be evaluated prospectively on other gemcitabine-treated PAAD patients.

## Introduction

Pancreatic cancer (PC) is the seventh leading cause of death among all cancers (495,773 new cases and 466,003 deaths worldwide in 2020 [1]). Surgical resection remains the only potential therapeutic option for patients with early-stage PC [2]. However, PC is difficult to diagnose due to nonspecific initial symptoms. Consequently, most patients are at the advanced or metastatic stage when they are diagnosed, where the 5-year survival rate is about 3% [3]. In addition, PC also lacks effective early detection methods, predictive biomarkers, and risk prediction models.

While several target therapies, chemotherapies, and immunotherapies have been approved for PC, gemcitabine has been widely used as the standard first-line treatment for PC. Gemcitabine is administered in combination with other cytotoxic agents, such as nab-paclitaxel, carboplatin, cisplatin, or oxaliplatin. Such gemcitabine-containing treatments generally offer enhanced patient survival and clinical benefit [4–10]. The anti-proliferative properties of gemcitabine come from inhibiting DNA synthesis [11]. Gemcitabine requires intracellular phosphorylation to be converted into its active form, difluorodeoxycytidine triphosphate (dFdCTP), which competes with deoxycytidine triphosphate (dCTP) for incorporation into DNA during DNA elongation, resulting in inhibition of DNA synthesis and inducing apoptosis of cancer cells [12]. However, 54% of gemcitabine-treated PC patients can exhibit primary resistance [13]. It is therefore important to find an effective way to anticipate which patients will benefit from gemcitabine. In this way, a more promising drug treatment could be administered without delay while avoiding gemcitabine’s adverse effects for patients who are unlikely to respond.

Several studies examined the gemcitabine-related biomarkers involved in its metabolism [14,15]. Most studies about predictive markers of gemcitabine treatment response focus on human equilibrative nucleoside transporter 1 (hENT1) expression, with high hENT1 expression being reported to show clinical benefit in selected treatment settings and survival endpoint [16–19]. However, some studies did not observe these benefits [20,21]. This might be due to study differences such as the way hENT1 expression is measured, different clinical endpoints, or different gemcitabine combos. This study will propose promising alternatives for this marker.

Computational models exploiting multi-omics and/or clinicopathological data to predict cancer patient outcomes have shown their value [22–27]. When the outcome is patient response to drugs, there is abundant preclinical pharmaco-omics data available [28]. These datasets have enabled drug response prediction via univariate markers [29,30] or gene expression signatures [31–33]. Machine learning (ML) algorithms, such as logistic regression, random forest, and deep neural network, have more recently been implemented to analyze such datasets and offer opportunities in personalized therapy to better understand mechanisms of drug resistance [34]. There are now a plethora of studies predicting drug response using ML trained on preclinical pharmaco-omics data [31,35–42]. Clinical pharmaco-omics datasets, despite being the most relevant for the patient, are much less available to train and evaluate ML models [43]. This is the reason for the small number [44–49].

ML techniques have been proven to be powerful tools to generate predictive computational models from the typically high-dimensional pharmaco-omics datasets. In particular, large-scale ML analysis, including algorithms enhancing supervised learning with feature selection such as optimal model complexity (OMC), has been shown to be advantageous [44,45,50]. To our knowledge, despite its promise, no study has yet applied a broad panel of ML algorithms to classify PC patients’ response to gemcitabine using various molecular profiles and clinical datasets. With this purpose, we retrospectively collected molecular profiles of The Cancer Genomic Atlas (TCGA)-Pancreatic Adenocarcinoma (PAAD) tumors from the U.S. National Cancer Institute (NCI) Genomic Data Commons (GDC) database (https://gdc.cancer.gov/). One of the objectives of GDC is generating standardized molecular profiles and clinical datasets across different cancer genome programs [51]. We span 8 molecular profiles ranking from 1,339 miRNA isoforms (isomiR) to the 450,000 DNA methylation of CpG probes and 8 ML classification algorithms that employed all features and OMC feature selection, producing a total of 128 diverse models to predict PAAD patients’ responses to gemcitabine. The goal of this study is to identify the robust ML model to classify gemcitabine responders and nonresponders in PAAD patients and to compare the predictive performances to gemcitabine single-gene marker.

## Methods

### Clinical data acquisition and preprocessing

The most common type of PC is PAAD [52]. The open access molecular profiling and clinical data of 185 primary pancreatic tumor samples in TCGA-PAAD project was downloaded from GDC application programming interface (API) (version 25.0). Neuroendocrine tumors and other noncancerous samples were thus not considered. To ensure the consistency of drug name, misspelling and synonyms (e.g., gemzar and gemcitabine HCl) of drugs were standardized according to the NCI drug dictionary and DrugBank to gemcitabine. Then, drug response information for each patient was obtained by querying the clinical records. The patients with missing gemcitabine response or responding inconsistently to a drug were excluded to retain only valid records. The patients who received gemcitabine prior to tumor resection were removed as indicated by the time of tumor procurement and the start of treatment. After these curation steps, 70 gemcitabine-treated PAAD patients remained. The gemcitabine responses provided by TCGA-PAAD were binarized into 2 classes: responder and nonresponder. We defined a responder as a patient who had complete response (CR) or partial response (PR), and a nonresponder as a patient who had stable disease (SD) or progressive disease (PD). TCGA-PAAD reports best response over the entire treatment period, starting from the initiation of the treatment until the end of treatment, i.e., not only the response observed at last follow-up.

### Molecular data acquisition and preprocessing

The curated gemcitabine responses were combined with the corresponding molecular profiles via TCGA patient IDs. Eight molecular profiles provided by GDC were considered in this analysis. The mRNA and miRNA expressions were generated from next-generation sequencing (NGS). mRNA(FPKM) was defined as mRNA in fragment per kilobase of exon per million mapped fragments (FPKM) unit, while mRNA(FPKM-UQ) was defined as mRNA in upper-quartile normalized FPKM unit. miRNA and isomiR were log_2_-transformed reads per million mapped reads (RPM). CpG (5′-cytosine-phosphate-guanine-3′) is beta values of DNA methylation level at known CpG sites using about 450,000 probes from Illumina Human Methylation 450 BeadChip. CGI is the average methylation beta of all probes’ values at known CpG sites corresponding to their CpG island. CNV(mean) is the average of copy number variations (CNVs) across segmented DNA corresponding to their gene using CNTools (version 3.8) of R package (version 3.5.1). CNV(median) is determined in the same way as CNV(mean), except the median is calculated. Those features missing any value across patients were removed. Thus, 8 datasets were generated, where each dataset was served as a set of features and subjected to ML classification algorithms to make the predictions. Note that we consider slightly different profile normalization to elucidate the impact of these differences on ML model performance.

### Data preparation for ML

**Fig. 1.**
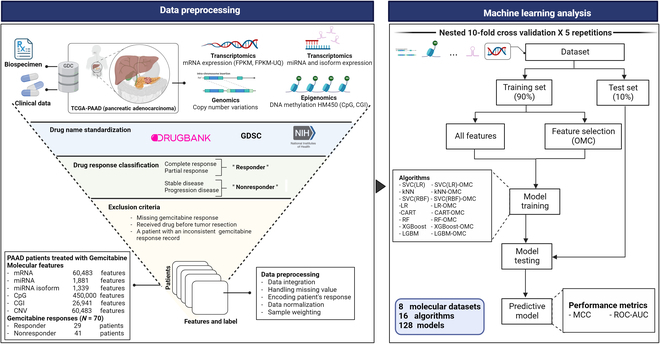
ML workflow to predict PAAD patient response to gemcitabine. The TCGA-PAAD clinical, biospecimen, and molecular profiles comprising mRNA in FPKM, and FPKM-UQ unit, miRNAs and isoforms, copy number variation, DNA methylation at CpGs, and CGI profiles from high-throughput technologies of patient tumors were retrieved from the NCI GDC. These molecular profiles were matched with corresponding clinical information using the patient’s TCGA barcode. The samples meeting the exclusion criteria were filtered out to retain only valid high-quality records. Each molecular profile of the tumors was preprocessed and subsequently submitted by each of the 8 supervised learning algorithm analyses. The performances of the resulting all-features ML models were evaluated by 10-fold CV (5 repetitions per model, each using a different random seed). Thus, 5 MCC and ROC-AUC determinations were carried out for each model. To mitigate the high dimensionality of the datasets, each of the 8 algorithms was coupled with the OMC strategy, with the resulting models undergoing identical performance evaluation. Overall, a total of 128 ML models (16 binary classification models for each of the 8 molecular profiles) were evaluated on the same cohort of patients. Further details can be found in the Methods section.

A computational framework was developed to estimate the predictive ability of molecular profiles on PAAD patients’ response to gemcitabine as shown in Fig. 1. In brief, each dataset was randomly split into a training and testing set using stratified *K*-fold, for *K* values of 5, 10, and 70. Eight ML classification algorithms, including classification and regression tree (CART), random forest (RF), extreme gradient boosting (XGBoost), light gradient boosting machine (LGBM), logistic regression (LR), support vector classifier (SVC) with linear kernel, SVC with the radial basis function (RBF) kernel, and *k*-nearest neighbors (KNN) algorithms, employed all features and OMC feature selection, producing a total of 128 models that were used to predict patients’ responses to gemcitabine. We performed 5 *K*-fold cross-validation (CV) repetitions, each with different random seed to assess the variability of resulting ML models. The median of evaluation metrics across 5 repetitions was reported. All analyses of this study were performed using Python version 3.7.3 (https://www.python.org/) and scikit-learn version 0.24.2 (https://scikit-learn.org).

### Building all-features classification models

Stratified *K*-fold standard CV runs were performed to estimate the predictive performance of ML algorithms for classification using all available features in each molecular dataset. After partitioning of the samples into *K*-folds, one of the *K*-folds was used as the test set for testing the model trained on the remaining *K* − 1 folds. The CV process was then repeated *K* times with each one of the *K*-folds used sequentially once in the test set. CV ensures that each patient has exactly one out-of-sample prediction regardless of the value of *K*. Model performance was not calculated on the test set of every fold and then averaged because each of the test sets would be too small and thus harm comparison to CV with other *K* values. As it is common with low-sample scenarios, we used instead a merged CV, where the 70 out-of-sample predictions were merged from all folds and Matthews correlation coefficient (MCC) was calculated once with all 70 samples.

### Building OMC classification models

The OMC models [45] were implemented in this study to overcome the high dimensionality problem, in this case, the number of features is larger than the number of patients. Stratified *K*-fold nested CV was performed to estimate how well classification ML algorithms perform using only the most relevant features to gemcitabine responses. In brief, the analysis of variance (ANOVA) test was used to calculate the *P* value of each feature, with a low *P* value indicating high discriminative power in terms of gemcitabine response. Then, classification algorithms were trained on only the considered subset of features (the top 2 to *n*/2 subset of features, where *n* is the number of samples). Among all *n*/2 trained models in the inner loop of the nested CV, the best model that achieved the highest MCC was selected and used to predict samples in its outer loop. The resulting 70 out-of-sample predictions were also merged from all folds, and each model performance metric was calculated once with all 70 samples.

### Building a model based on hENT1 expression

hENT1 has been identified as a prognostic molecular marker in PC patients treated with gemcitabine [16,17]. To compare the performance of the best models to that of using a single hENT1 gene only, hENT1 expression derived from the mRNA(FPKM) profile was trained on the same algorithms as the 2 most predictive models (RF and XGBoost) using standard 10-fold CV from merging the out-of-sample predictions from the 10 folds.

### Model performance evaluation

When a model estimated real-valued class probabilities, these were transformed to binary classes by setting a threshold to 0.5. The probabilities above this threshold were classed as responders; otherwise, they were classed as nonresponders. The 4 confusion matrix categories, including true positives (TP), true negatives (TN), false positives (FP), and false negatives (FN), were counted and used to calculate performance metrics including MCC and receiver operating characteristic–area under the curve (ROC-AUC).

### Construction of protein–protein interaction (PPI) network

To unravel the relationships among the genes corresponding to 12 predictive CpG probes, these genes were mapped to STRING database version 11.5 (https://string-db.org/) [53] to retrieve both known and predicted protein–protein interactions (PPIs). We limited the species to “Homo sapiens” and required at least the minimum confidence interaction score (combine score > 0.15). The text mining, experiments, database, co-expression, co-occurrence, neighborhood, and gene fusion evidences were included to construct PPI.

### The pathway enrichment analysis

In order to understand the roles of the genes found to be associated with the predictive CpG probes in PAAD treated with gemcitabine, the genes corresponding to 12 CpG probes were subjected to the Database for Annotation, Visualization and Integrated Discovery (DAVID) web server [54] for Gene Ontology (GO) pathway enrichment analysis. In addition, the Kyoto Encyclopedia of Genes and Genomes (KEGG) pathway enrichment analysis was also investigated using the KEGG Orthology-Based Annotation System (KOBAS) web server (http://kobas.cbi.pku.edu.cn/) [55]. A statistically significant *P* value was calculated using a Fisher exact test, which further employed the Benjamini–Hochberg multiple-testing correction for the correct *P* value. The significant enriched pathways were identified based on a *P* value less than 0.05. For each pathway, input genes that are part of the pathway are counted, and the enrichment ratio was also calculated.

### Statistical analysis

The two-tailed unpaired Welch’s *t* test was used to generate *P* values in data analysis. The median overall survival (OS) and progression-free survival (PFS) were calculated for 4 groups, including actual and predicted responders and nonresponders, representing the time point on the Kaplan–Meier plot where 50% of the patients in each group have survived (for OS) or have not experienced disease progression (for PFS). Log-rank test was used to compute statistical differences in OS and PFS. The univariate and multivariate Cox regression analyses were performed to assess hazard ratio (HR) and 95% confidence intervals (CIs) of gemcitabine response predictors (4 mRNAs and 12 CpG probes). A *P* value less than 0.05 was considered as statistically significant.

## Results

A total of 70 molecularly profiled PAAD patients treated with gemcitabine (41 nonresponders and 29 responders) were derived from TCGA-PAAD project. The patient’s distribution and available features of each molecular profile, spanning from the 1,339 miRNA isoform expressions to the 450,000 methylation levels of DNA probes, were presented in Fig. S1. We trained each of 8 molecular profiles from PAAD patients’ tumor on 8 classification ML algorithms with and without OMC feature selection, producing a total of 128 gemcitabine response prediction models. Computational pipeline is described in the Methods section and is represented in Fig. 1.

### ML model performances at predicting PAAD patient response to gemcitabine

**Fig. 2.**
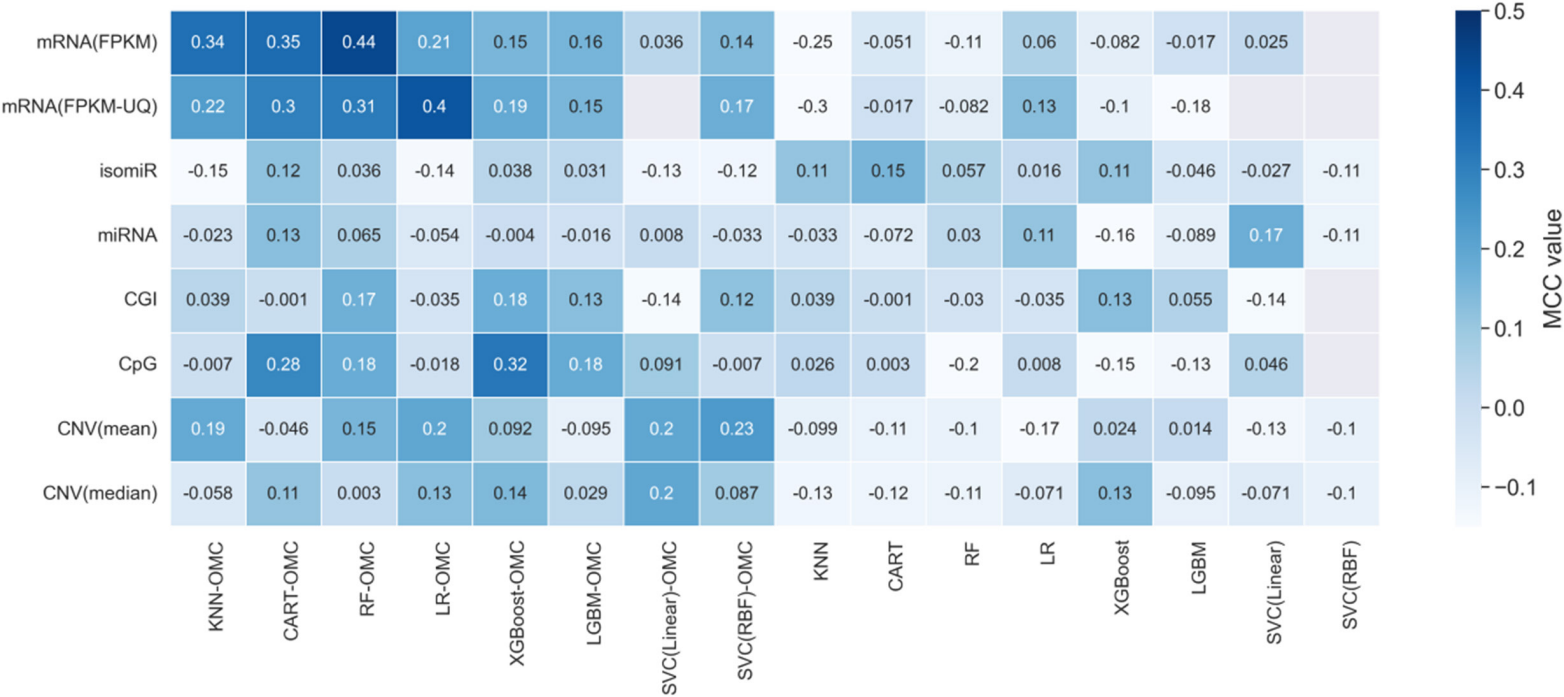
Heatmap presenting the mMCC of the five 10-fold CV repetitions for each of the 128 models. The vertical axis shows the considered molecular profiles, while the horizontal axis shows the ML algorithms used. The first 8 columns on the left show supervised ML algorithms implemented with OMC feature selection considering only the subset of informative features during the model training (the suffix “OMC” was added to the algorithm name), while the remaining 8 columns on the right show the corresponding algorithms that used all features available in datasets. Five MCCs were obtained from five 10-fold CV runs ranking from −1 to 1. The mMCC was shown in the heatmap. A positive MCC means that the model accurately predicts gemcitabine responses better than a random classifier. MCC of 0.0 indicates a prediction no better than a random. A negative MCC means that the model performs worse than a random. A model that obtained undefined MCC, predicting the same class for all instances where the denominator of MCC is zero, is indicated as blank boxes. The 2 most predictive molecular profiles were mRNA expression and DNA methylation of CpG: mMCC of 0.44 from RF-OMC using mRNA expression profiles and mMCC of 0.32 from XGBoost-OMC using DNA methylation of CpG profile. In addition, the OMC models tend to perform better than the corresponding all-features models.

For each of the algorithm–profile pair, the model performance was evaluated by stratified 10-fold CV with 5 repetitions, each repetition with a different random seed. Figure 2 shows the out-of-sample median MCC (mMCC) of the 5 repetitions (the boxplot with these 5 MCC determinations can be seen in Fig. S2). OMC models, which are enhanced with further feature selection, tend to perform better than all-features models. Indeed, only 31 (48.4%) of the 64 OMC models yield mMCC values below 0.1, compared to 56 (87.5%) of the all-features models. Models exploiting mRNA and DNA methylation profiles distinguish responders from nonresponders with mMCCs of over 0.3. Both mRNA(FPKM) and mRNA(FPKM-UQ) exhibited the similar predictive performances regardless of normalized gene expression metrics used. mRNA(FPKM) achieved the highest MCC; hence, it is recommended for this challenging problem. RF-OMC and LR-OMC incorporating mRNAs are the 2 best ML models, achieving mMCC of 0.44, and 0.4, respectively. In addition, XGBoost incorporating DNA methylation of CpG probes obtained mMCC of 0.32. Such poor performance was also observed when using SVC for these problem instances. All nonresponders were misclassified using some molecular profiles [either mRNA(FPKM), mRNA(FPKM-UQ), CpG, or CGI], leading to undefined MCCs. Median ROC-AUCs range from 0.407 to 0.674 (Fig. 2).

### OMC models are generally more predictive while retaining a concise subset of predictive features

**Fig. 3.**
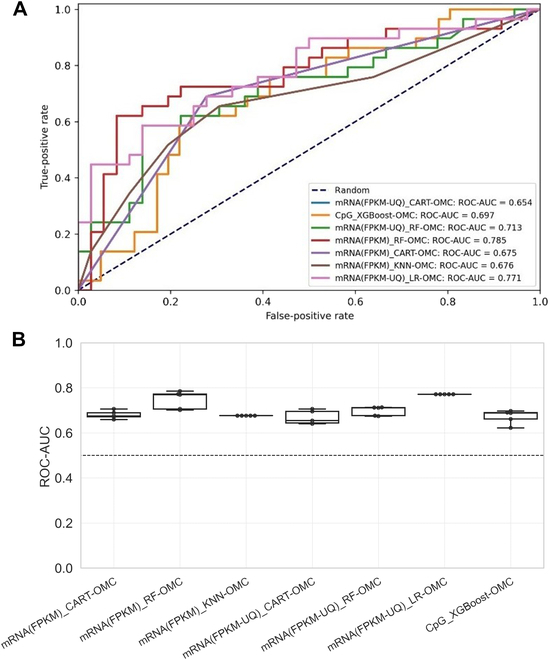
ROC-AUC across five 10-fold CV runs of the 7 most predictive models with the highest median ROC-AUC. (A) Highest ROC-AUC among five 10-fold CV runs for each model. A ROC curve depicts the trade-off between the true-positive rates and the false-positive rates of a classifier by varying the threshold of the probability of response. A ROC-AUC of 1 is the best model performance at distinguishing between responders and nonresponders, while a ROC-AUC of 0.5 corresponds to the random guessing denoted by a dashed line. RF-OMC that employed the mRNA model has the highest ROC-AUC of 0.785. (B) ROC-AUC variability across the five 10-fold CV runs for each of the 7 most predictive models with the highest median ROC-AUC. The horizontal dashed line at 0.5 represents random-level performance.

OMC is the process of selecting the most informative features that considerably reduces the number of features for model building. As shown in Fig. 2, the OMC models tend to be more predictive, whereas the all-features models obtained a near-random predictive level. The most predictive models with an mMCC of at least 0.3 were OMC models that employed either mRNAs or CpG probes (Fig. S3). These 7 predictive OMC models also obtained the highest median ROC-AUCs in this challenging problem, ranking from 0.654 with CART-OMC employing mRNA(FPKM) to 0.785 with RF-OMC employing mRNA(FPKM) (Fig. 3). We employ MCC as the main evaluation metric, as it is less flattering than ROC-AUC.

### The differential expression of the gemcitabine predictors

**Fig. 4.**
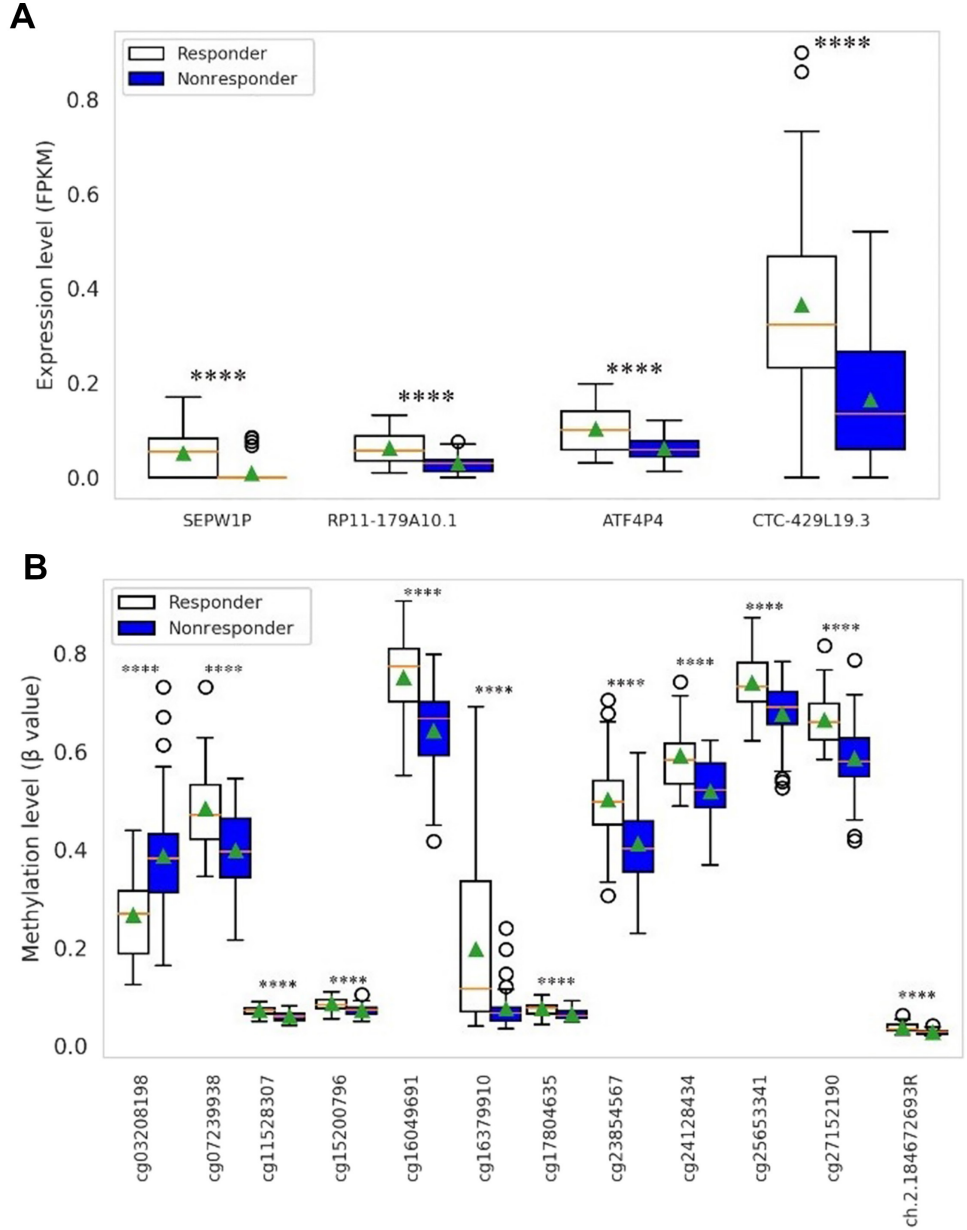
Boxplot showing the differential expression of the predictive genes and DNA methylation of CpG probes between responders and nonresponders. The *y* axis represents the normalized expression level for 4 predictive genes from RF-OMC employing mRNA(FPKM) profile (A) and 12 predictive DNA methylation CpG probes from XGBoost-OMC employing CpG profile (B) between responders and nonresponders. The corresponding gene(s) for each CpG probe can be found in Table S6. The orange line inside each boxplot represents the median expression across patients for a given feature, while the green triangle represents the mean expression. The boxplot for each feature contains the *P* value of mean differential expression between responders and nonresponders using a two-sided Welch’s *t* test. ****P ≤ 0.0001, significant difference in expression levels between 2 groups.

mRNA expression is the most predictive profile for stratifying PAAD patients treated with gemcitabine, giving the highest MCC. Four (SEPW1P, RP11-179A10.1, ATF4P4, and CTC-429L19.3) of 60,483 mRNAs are the most predictive features selected by RF-OMC that employed mRNA(FPKM) profile (mMCC of 0.44). Figure 4 and Table S2 show the differential expression of these 4 predictive genes between the responders and nonresponders using two-sided Welch’s *t* test. All these 4 predictive genes are significantly (*P* < 0.01) up-regulated in responders compared to nonresponders. CTC-429L19.3 is the most expressed genes, while ATF4P4, RP11-179A10.1, and SEPW1P are the least expressed genes. Interestingly, these 4 predictive genes were commonly selected by other 5 most predictive OMC models with an mMCC of at least 0.3 employing mRNA profiles (Table S3). In particular, CART-OMC, the second most predictive model that employed mRNA(FPKM), also combined the same 4 predictive mRNAs to build classification tree (mMCC of 0.35) (Fig. S4).

While 12 of 450,000 CpG probes [cg03208198 (COL18A1 and MIR6815), cg07239938 (ELANE), cg11528307 (C14orf80 and CRIP1), cg15200796 (TMEM191C), cg16049691 (AHRR), cg16379910 (B2M), cg17804635 (ZNF703), cg23854567 (PXN), cg24128434 (DNAH2), cg25653341 (PLOD3), cg27152190 (RP11-429P3.3), and ch.2.184672693R] were selected as predictive features by XGBoost-OMC that employed DNA methylation of CpG profile, the genes found to be related to each of these probes are between the brackets except for ch.2.184672693R for which GDC did not report any associated genes. Two-sided Welch’s *t* tests show that the responders had significantly (*P* < 0.01) higher DNA methylation of these predictive CpG probes than nonresponders, except cg03208198, which showed lower DNA methylation in responders (Fig. 4 and Table S2). In addition, the feature importance for the top 2 best models was shown in Fig. S10. It has been found that ENSG00000267197.1 was selected by RF-OMC model as the most important feature in predicting gemcitabine response (Fig. S10A), while cg27152190 is the most importance feature for XGBoost-OMC model prediction (Fig. S10B).

### PPI network analysis of genes corresponding to the 12 predictive CpG probes

**Fig. 5.**
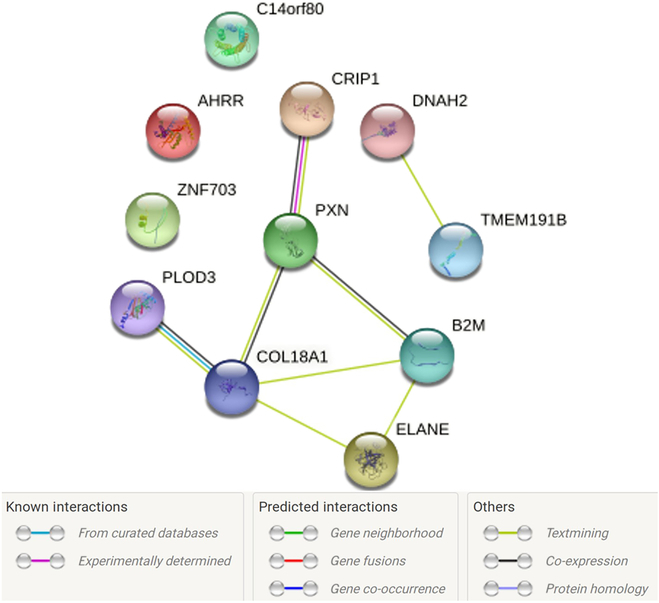
The PPI network analysis. The genes associated with the 12 predictive CpG probes (Table S8), as annotated by the GDC, were input into STRING database for PPI network analysis. Out of 13 genes corresponding to predictive CpG probes, 11 protein-coding genes were found in the STRING database, while the remaining 2 genes (MIR6815 and RP11-429P3) were not found as they are non-protein-coding genes (MIR6815 is miRNA, and RP11-429P3.3 is antisense). These 11 protein-coding genes were connected by 14 edges representing their known and predicted interactions as shown in the legend. A PPI enrichment *P* value of 0.035 was obtained, indicating that the protein input has more interactions among themselves than what would be expected for a set of proteins of the same size and degree distribution drawn at random from the genome. As a result, the proteins were connected as a group. The nodes in the PPI network represent proteins, and the edges represent their known and predicted interactions.

The interactions among the genes corresponding to 12 predictive CpG probes were assessed using STRING web server [53], which integrates both known and predicted PPIs. Deciphering the protein interaction network could help in understanding the physical and functional associations of these protein that could influence chemo-susceptibility. Eleven of 13 genes corresponding to predictive CpG probes were mapped in the STRING database, while the remaining 2 non-protein-coding genes were not mapped (MIR6815 is miRNA, and RP11-429P3.3 is antisense). Figure 5 shows the network with 11 nodes and 14 edges of PPI. The nodes are proteins, while edges are their interactions (PPI enrichment *P* value of 0.035). The known and predicted interactions were observed among 6 proteins (CRIP1, PXN, COL18A1, B2M, ELANE, and PLOD3). TMEM191B and DNAH2 were linked with text mining of scientific literature evidence. The remaining 3 genes (C14orf80, AHRR, and ZNF703) were disconnected nodes in the network. These 13 genes corresponding to predictive CpG probes were also enriched in cancer-associated pathways in GO and KEGG pathway enrichment analysis (see the next section). Overall, the results show that these gene interactions could contribute to the gemcitabine resistance in PAAD patients.

An analogous analysis could not be performed with the 4 predictive mRNAs, as these do not code for proteins and hence are not included in the STRING database. Two of them (ATF4P4 and SEPW1P) are annotated as pseudogenes, while the other 2 (RP11-179A10.1 and CTC-429L19.3) are long noncoding RNAs.

### GO and KEGG pathway enrichment analysis of genes corresponding to the 12 predictive CpG probes

The GO enrichment analysis of genes corresponding to 12 predictive CpG probes revealed a total of 43 significantly (*P* < 0.05) enriched GO pathways (Fig. S5 and Table S4) across 3 GO categories: biological process (BP), cellular component (CC), and molecular function (MF). By analyzing BP, we found that genes were enriched in 39 GO BP pathways. Most of these pathways are cancer-related pathways and contributed to cancer progression, for example, cell differentiation and development, cell proliferation, regulation of cell adhesion, and cell motility. Cancer cells undergo rapid proliferation and migration [56]. Cellular adhesion is also an important process in cancer progression, as it allows cancer cells to interact with and invade surrounding tissue [57]. Additionally, the extracellular matrix and extracellular structures are important components of the tumor microenvironment and play a critical role in cancer progression [58]. These pathways provide insights into the underlying biological mechanisms that drive cancer development. Interestingly, 8 genes (COL18A1, PXN, ZNF703, AHRR, PLOD3, CRIP1, B2M, and ELANE) were predominantly enriched in response to chemical and response to stimulus pathways. Dysregulation of these 8 enriched genes in response to chemical or stimulus pathways could promote carcinogenesis and drug resistance [59–61]. Six of these 8 enriched genes were also linked with known and predicted interaction evidences in PPI network, suggesting that these genes may function in coordinated manner and contributed to drug resistance (Fig. 5). Furthermore, GO enrichment through CC showed that the terms endoplasmic reticulum lumen, macromolecular complex, and specific granule lumen indicate an intracellular localization where they are likely to interact. By analyzing MF, 2 genes were enriched in transcriptional repressor activity and RNA polymerase II transcription factor binding pathways. This pathway was related to gemcitabine mechanism, which was involved in negative regulation of transcription from an RNA polymerase II promoter that led to apoptosis [62].

The KEGG pathway enrichment analysis (Table S5) showed that genes corresponding to CpG probes were significantly (*P* < 0.05) enriched in 10 KEGG pathways, with the metabolism of xenobiotics by cytochrome P450 pathway being particularly noteworthy due to its involvement in drug metabolism and patient response to treatment. Overexpression of cytochrome P450 causes rapid drug elimination before it reaches the target site [63]. Moreover, antigen processing and presentation pathway is critical for the recognition of tumor cells by the immune system. Tumor cells can present antigens on their surface, which can be recognized by T cells and trigger an immune response. Immunotherapy approaches such as immune checkpoint inhibitors target this pathway to enhance T cell activity and improve antitumor immunity [64].

Altogether, our findings show that dysregulation of DNA methylation of predictive CpGs in this study could be associated to the alteration of their corresponding genes and pathways in which they are involved, leading to cancer development and gemcitabine resistance in PAAD patients. The pathway enrichment analysis provides insight into the underlying biological mechanisms of genes corresponding to predictive CpG that could be targeted for cancer therapy. However, functional validations are needed to test our bioinformatics findings.

### Assessing the robustness of the best model in predicting PAAD patients’ gemcitabine responses

**Fig. 6.**
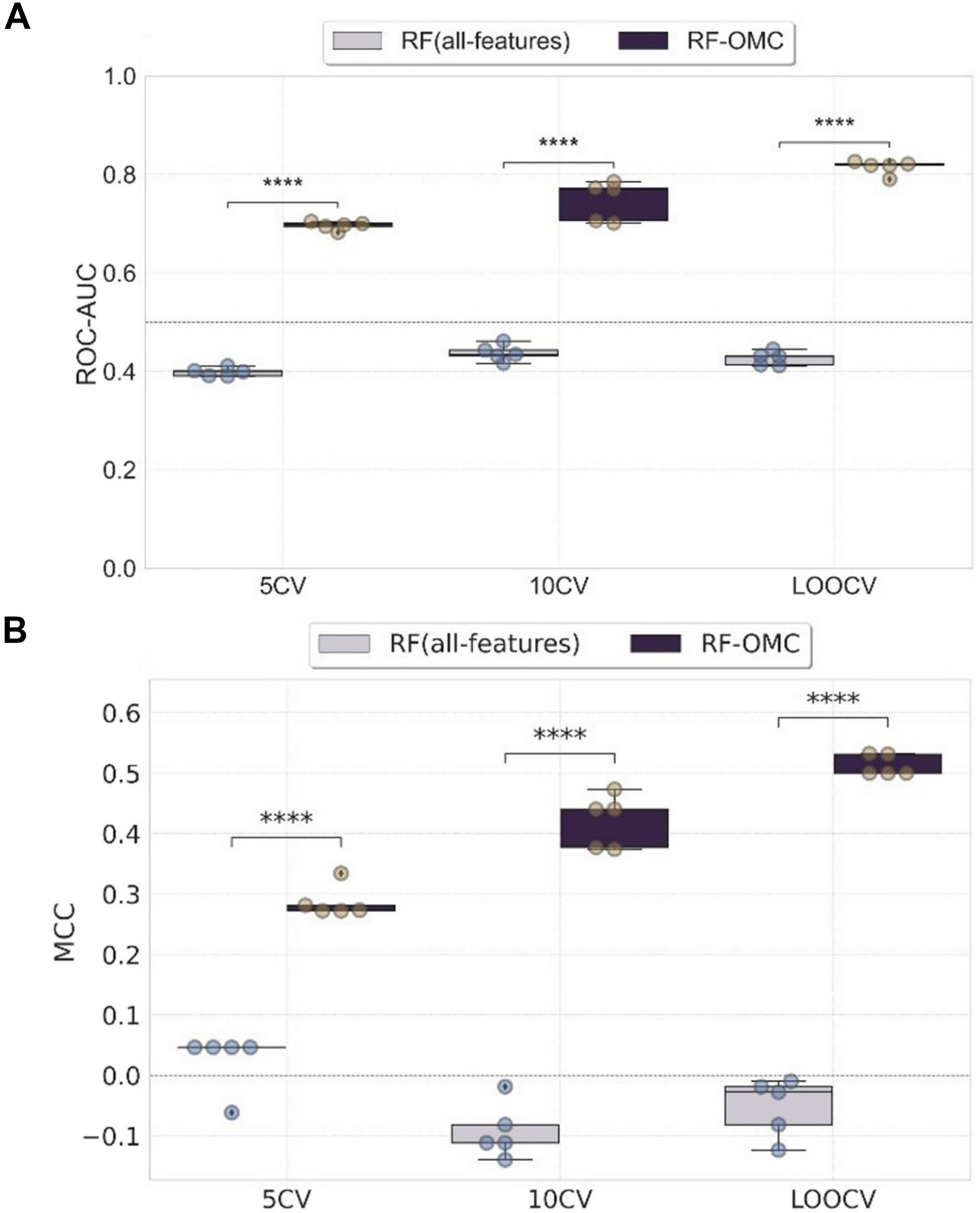
The impact of training dataset size on the performance of best model versus its corresponding all-features model. The boxplots present the distributions of MCC (A) and ROC-AUC (B) obtained across five 10-fold CV runs of RF-OMC employing mRNA(FPKM) profile with 5CV, 10CV, and LOOCV (i.e., *K* = 5, 10, and 70, respectively). RF incorporated OMC feature selection (RF-OMC) was represented in deep purple, while RF trained on all available 60,483 mRNA features [RF(all-features)] was represented in light purple. Each boxplot contains the *P* value of mean differential expression between 2 groups with a two-sided Welch’s *t* test. ****P ≤ 0.0001. The model performances improved as the training dataset size increased. In addition, the OMC models significantly outperformed the corresponding all-features models in all CVs. The dashed line represents the random classifier at MCC of 0 and ROC-AUC of 0.5. MCC or ROC-AUC of 1 indicates a perfectly accurate prediction.

We next investigated the effect of training dataset sizes on the predictive performance of the best model. Leave-one-out CV (LOOCV), 5-fold CV (5CV), and 10-fold CV (10CV) were used to evaluate the RF-OMC that employed mRNA(FPKM) profile model performance. All the CVs were repeated exactly in the same manner for all-features models. The MCCs and ROC-AUCs of 5 repetitions, each with different random seeds, are presented in Fig. 6. The results show the improvement in predictive performances as we increase the training dataset size from 80% of the data in 5CV to 99% of the data in LOOCV, while all-features models obtained a near-random predictive level and were significantly worse than those obtained from OMC models in all cases (Fig. 6). We also investigated whether the performances obtained from the signal in the dataset was better than what would be predicted by chance from class-permuted version. As a result, all permutation models were significantly worse than those arising from OMC models in all cases (Fig. S6). The similar result was obtained when using XGBoost-OMC, which employed DNA methylation of CpG profile and showed improved predictive performance as the training dataset size increased (Fig. S9).

### CpG-based XGBoost-OMC can predict survival of gemcitabine-treated PAAD patients

**Fig. 7.**
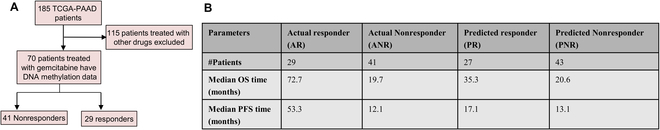
Comparison between the median OS and PFS time of actual patient response to gemcitabine and those predicted by the CpG-based XGBoost-OMC model. (A) Flowchart of patient selection. (B) Median OS (defined as the time interval from the date of diagnosis to death or the last known follow-up date) and PFS (defined as the time interval from the date of diagnosis to disease progression or the last known follow-up date) time for 4 groups, including actual and predicted responders and nonresponders by the XGBoost-OMC model. The median OS and PFS are defined as the time point on the Kaplan–Meier plot, where 50% of the patients in each group have survived (for OS) or have not experienced disease progression (for PFS). The figure highlights the ability of the CpG-based XGBoost-OMC model to accurately predict patient gemcitabine response, as demonstrated by the similar trend between the predicted and actual outcomes. The actual and predicted responders had longer OS and PFS than actual and predicted nonresponders.

We evaluated the OS (defined as the time interval from the date of diagnosis to death or the last known follow-up date) and PFS (defined as the time interval from the date of diagnosis to disease progression or the last known follow-up date) time difference between actual and predicted responders and nonresponders by the XGBoost-OMC combined 12 CpG probes. Among 70 patients with survival data and gemcitabine responses, 41 (58.6%) nonresponders and 29 (41.4%) responders (Fig. 7A), XGBoost-OMC incorporating 12 CpG probes classified these patients into 43 predicted nonresponders and 27 predicted responders. The confusion matrix was shown in Table S3.

The median OS and PFS were calculated for 4 groups, including actual and predicted responders and nonresponders by the XGBoost-OMC model. The median OS and PFS were not significantly different between the actual and the predicted responders (log-rank test *P* value of 0.19 for OS and 0.14 for PFS) and nonresponders (log-rank *P* value of 0.33 for OS and 0.11 for PFS). The actual responders had significantly longer OS (median OS was 72.7 months) and PFS (median PFS was 53.3 months) than actual nonresponders (median OS was 19.7 months; median PFS was 12.1) with log-rank test *P* value < 0.005. Similarly, the predicted responders had longer OS (median OS was 35.3 months) and PFS (median PFS was 17.1 months) compared to the predicted nonresponders (median OS was 20.6 moths; median PFS was 13.1). However, the difference was not statistically significant with log-rank test *P* value of 0.19 and 0.09 in OS and PFS, respectively (Fig. 7B).

### mRNA-based RF-OMC can also predict survival of gemcitabine-treated PAAD patients

**Fig. 8.**
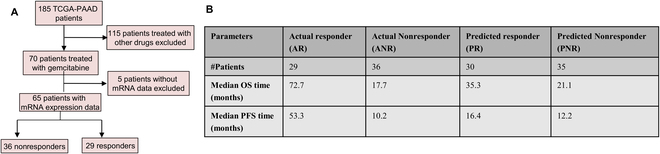
Comparison between the median OS and PFS time of actual patient response to gemcitabine and those predicted by the mRNA-based RF-OMC model. (A) Flowchart of patient selection. (B) Median OS (defined as the time interval from the date of diagnosis to death or the last known follow-up date) and PFS (defined as the time interval from the date of diagnosis to disease progression or the last known follow-up date) time for 4 groups, including actual and predicted responders and nonresponders by the RF-OMC model. The median OS and PFS are defined as the time point on the Kaplan–Meier plot where 50% of the patients in each group have survived (for OS) or have not experienced disease progression (for PFS). The figure highlights the ability of the mRNA-based RF-OMC model to accurately predict patient gemcitabine response, as demonstrated by the similar trend between the predicted and actual outcomes. The actual and predicted responders had longer OS and PFS than actual and predicted nonresponders.

Among 65 mRNA profiled patients, 36 nonresponders and 29 responders (Fig. 8A), RF-OMC incorporating 4 mRNAs classified these patients into 35 predicted nonresponders and 30 predicted responders. The confusion matrix was shown in Table S3. The median OS and PFS were not significantly different between the actual and the predicted responders (log-rank test *P* value of 0.21 for OS and 0.15 for PFS) and nonresponders (log-rank *P* value of 0.45 for OS and 0.20 for PFS). The OS and the PFS were significantly longer in the actual responders (median OS was 72.7 months; median PFS was 53.3 months) compared to the actual nonresponders (median OS was 17.7 months; median PFS was 10.2 months) with log-rank test *P* value < 0.005. Similarly, the OS and PFS were longer in the predicted responders (median OS was 35.3; median PFS was 16.4) compared to the predicted nonresponders (median OS was 21.1; median PFS was 12.2) with log-rank test *P* value of 0.19 for OS and 0.04 for PFS (Fig. 8B).

Notably, the median OS and PFS time of actual response to gemcitabine and those predicted by CpG-based XGBoost and mRNA-based RF-OMC models exhibited the similar results. The median PFS of predicted responders and predicted nonresponders by the mRNA-based RF-OMC model shows a statistically significant difference (log-rank test *P* value of 0.04), despite the small sample size (Fig. 8B). This suggests that RF-OMC combining 4 mRNAs could be a prognostic predictor of gemcitabine-treated PC patients.

### Identification of the predictive 4 mRNAs and the 12 CpG probes as the independent survival predictors in gemcitabine-treated PAAD patients

We next investigated whether gemcitabine response predictors (predictive 4 mRNAs and 12 CpG probes) could be survival predictors in PAAD patients. The univariate Cox regression analysis of 4 mRNAs from 65 mRNA profiled patients revealed that 3 genes could be independent biomarkers for prediction of OS and PFS (*P* < 0.05) (Fig. S7A). The high expression of SEPW1P (HR = 0.59 for OS; HR = 0.72 for PFS), RP11-179A10.1 (HR = 0.63 for OS; HR = 0.58 for PFS), and CTC-429L19.3 (HR = 0.64 for OS; HR = 0.58 for PFS) was significantly associated with improved OS and PFS, but it was not significant in multivariate Cox regression analysis (Fig. S7, A and C). These finding were consistent with the differential expression of these 4 mRNAs, which were shown to be highly expressed in responders (Fig. 4).

Furthermore, the univariate analysis of the 12 CpG probes from 70 DNA methylation profiled patients revealed that 3 CpG probes could be used as independent biomarkers for OS prediction (*P* < 0.05) (Fig. S7B). The high DNA methylation of cg15200796 (HR = 0.68) and cg16049691 (HR = 0.7) was significantly associated with improved OS. In contrast, high DNA methylation of cg03208198 (HR = 1.4) was significantly associated with poor OS. In addition, 4 CpG probes could be used as independent biomarkers to predict PFS (*P* < 0.05) (Fig. S7B). The high DNA methylation of cg07239938 (HR = 0.71), cg15200796 (HR = 0.77), and ch.2.184672693R (HR = 0.61) was associated with improved PFS, whereas high DNA methylation of cg03208198 (HR = 1.47) was negatively associated with PFS. The remaining CpG probes had no significant association with patient survival. In multivariate Cox regression analysis, only cg03208198 (HR = 1.56 for OS; HR = 1.41 for PFS) was shown to be significantly associated with OS and PFS (Fig. S7D). These results were consistent with the differential expression of these 12 CpG probes, where high DNA methylation of cg03208198 was associated with nonresponders, while the remaining CpG probes showed high DNA methylation in responders (Fig. 4). These suggest that gemcitabine response predictors could be used as independent survival predictors of PAAD patients receiving gemcitabine.

Subsequently, we also performed univariate Cox regression analysis on hENT1 gene. The result in Fig. S8A shows that hENT1 gene was not significantly associated with both OS (HR = 1.01; *P* = 0.63) and PFS (HR = 1.0; *P* = 0.76) in the univariate model, suggesting that hENT1 gene was not prognostic in PAAD patient receiving gemcitabine. In addition, Fig. S8B demonstrates poor patient stratification by using hENT1 gene trained on RF model, in which there was a trend toward better survival in predicted nonresponders.

### The effect of data integration on the gemcitabine response prediction

**Fig. 9.**
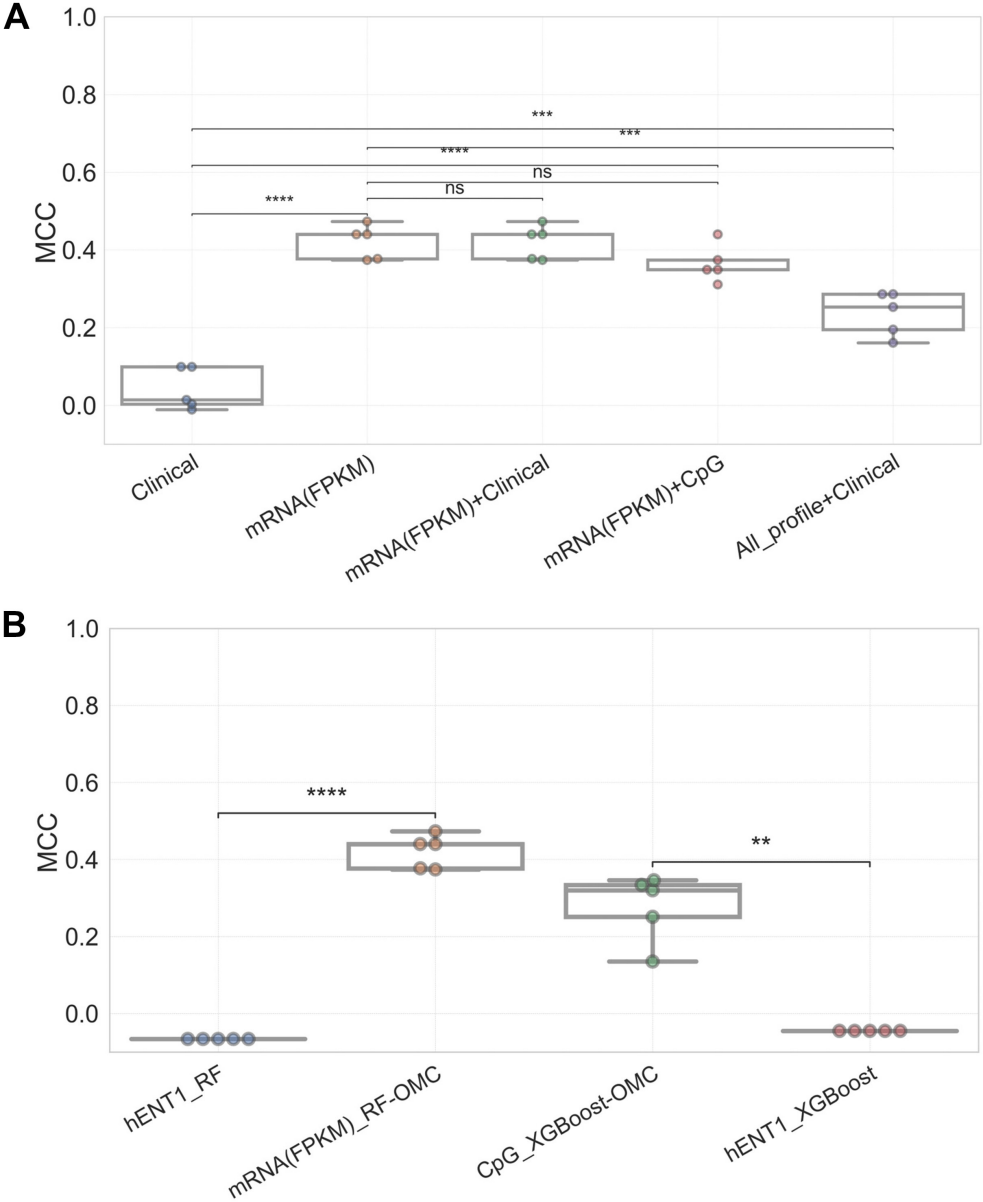
Comparison of the predictive performances of the best model to those combining other datasets and those using hENT1 single gene only in predicting gemcitabine response in PAAD patients. (A) Boxplots comparing the MCCs obtained from five 10-fold CV runs (each dot represents a run) of the RF-OMC prediction based on mRNA profile [mRNA(FPKM)], combining mRNA and clinical dataset [mRNA(FPKM) + Clinical], combining mRNA and CpG profile [mRNA(FPKM) + CpG] and clinical data only (Clinical) (Table S1), and combining all of 8 molecular profiles and clinical data (All_profile + Clinical). MCC ranges between −1 (misclassification) and 1 (perfect classification), with 0 corresponding to no better than a random. Altogether, integrated patients’ clinical data have no effect on the predictive performance of the best model. Importantly, increasing number of features could reduce the model performances. (B) To compare the predictive performances of the 2 most predictive models with those using hENT1 only, hENT1 gene expression from mRNA(FPKM) profile was trained on the same algorithms as the 2 most predictive models (RF and XGBoost). Boxplots comparing the MCCs of the 2 most predictive models [mRNA(FPKM)_RF-OMC and CpG_XGBoost-OMC] to those using the expression of the hENT1 gene only (hENT1_RF and hENT1_XGBoost) in gemcitabine response prediction of PAAD patients. The 2 most predictive models obtained in this study significantly outperformed the prediction based on hENT1 expression as a single predictor. Statistical comparisons between different models were performed using two-sided Welch’s *t* test. Stars denote the *P* value, where nonsignificant “ns” means 0.05 < *P* ≤ 1.00. *0.01 < *P* ≤ 0.05, **0.001 < *P* ≤ 0.01, ***0.0001 < *P* ≤ 0.001, and ****P ≤ 0.0001.

We next investigated the effect of data integration on the predictive performance of the best model. The RF-OMC was trained on mRNA profile, clinical features (Table S1), and integrated mRNA and clinical features. Figure 9A shows the MCCs across five 10-fold CV repetitions. Combining mRNA and clinical features (FPKM + clinical) did not improve the model performance (mMCC of 0.44) compared to those built on only mRNA features (FPKM) (mMCC of 0.44). RF-OMC prediction based on only clinical data (clinical) led to the lowest performance (mMCC of 0.014). If merging the 2 best molecular profiles provided a performance boost, it would justify the time and financial overhead of determining both profiles in every patient. However, RF-OMC integrating the 2 most predictive CpG methylation and mRNA profile performed worse than those by individual profile (mMCC of 0.349). Last, RF-OMC employed all the molecular profiles and clinical features (All_profiles) did not improve the prediction (mMCC of 0.253). Overall, there is no benefit in integrating patients’ clinical data with molecular profiles on the predictive performance of the best model. Importantly, increasing number of features could reduce the model performances as a curse of dimensionality. We did not carry out systematic ablation experiments combining more than 2 profiles mostly due to unfavorable cost–benefit reasons.

### Comparing the model prediction based on mRNA and CpG methylation profiles to the hENT1, a well-established gemcitabine response biomarker

Many studies reported that hENT1 has shown to be a predictive biomarker of gemcitabine response in PC. The high expression of hENT1 is associated with sensitivity to gemcitabine and increased overall and disease-free survival [16,17,65]. Figure 9B shows that RF-OMC combining 4 mRNAs (mMCC of 0.44) significantly outperformed RF prediction using only hENT1 gene expression (mMCC of −0.066). Similarly, XGBoost-OMC combining 12 CpG probes (mMCC of 0.32) obtained a significantly higher MCC compared to XGBoost that employed only hENT1 gene expression (mMCC of −0.045). Table S6 also presents the predictive performances in terms of MCC and ROC-AUC. The results suggest that the prediction based on either 4 predictive mRNAs or 12 CpG probes outperforms those by hENT1 single-gene biomarker in predicting gemcitabine responses.

## Discussion

Omics-based precision oncology, also known as targeted therapies or genomic medicine, has improved cancer patient management. The focus has been on either single-gene marker [17,66,67], a single molecular profile [67–70], or the discovery of rare actionable mutations [71,72]. However, predicting drug responses is a challenging task in oncology. Using a single-gene marker or a few molecular profiles is often insufficient to correctly predict drug responses [50,73,74]. The previous researches [44,50,75] demonstrated that the multiple molecular profiles provide new opportunities to identify robust biosignatures that can discriminate drug responses and aid to tailor treatments for individual cancer patients [44,50,75].

Supervised ML algorithms are increasingly being applied to the multiple molecular profiles as they can predict the clinical outcomes for new data based on what they learned from the previous data. Owing to well-curated molecular profiling data of GDC, we present a large-scale analysis of supervised 128 ML models built on multiple molecular profiles and algorithms to predict gemcitabine responses in 70 PAAD patients. Smaller sample sizes are not uncommon for cancer-specific drug response prediction problems [76]. Here, out-of-sample predictions were carried out with rigorous CV procedures, which have been shown to anticipate held-out test set performance in smaller datasets than the one used in our study [77].

Using five 10-fold CV runs, 7 of 128 (5%) models are predictive with mMCC > 0.3. These 7 predictive models were all OMC models. Training only the most informative features in OMC models increases the chance of making accurate prediction by removing the irrelevant features from high dimensionality dataset early prior to model training, and model performance improves compared to all-features models. The 2 most predictive molecular profiles in this study revealed 4 of 60,483 mRNAs (0.066%) from RF-OMC model and 12 of 450,000 CpG probes (0.027%) from XGBoost-OMC model as the most informative biosignatures for predicting gemcitabine responses in PAAD patients. These models did not only exhibit high out-of-sample mMCC values robust to different partitions and repetitions, but these values were also significantly higher than those from all-features models (Fig. 6) and class-permuted models (Figs. S6 and S9). Moreover, the performance of these 2 OMC models monotonically increased with larger training set sizes (Fig. 6). Last, note that, in general, other combinations of molecular profiles and algorithms will be predictive in different drugs and cancer types [44,45,50].

The RF-OMC model nonlinearly combined 4 predictive mRNA features including SEPW1P, RP11-179A10.1, ATF4P4, and CTC-429L19.3 that have accurately predicted PAAD patients’ responses to gemcitabine. Two of these predictive mRNAs (ATF4P4 and SEPW1P) are pseudogene, while the remaining mRNAs are RNA genes (RP11-179A10.1 and CTC-429L19.3) (Table S7). These genes might be involved in drug resistance and cancer progression through alterations in their expression. Other studies have already examined the roles of these genes in other types of cancer. For example, depletion of SEPW1P enhances tumor suppressor activities in breast cancer [78]. The single-nucleotide polymorphisms (SNPs) located in long noncoding RNA RP11–179A10.1 were reportedly associated with poorer outcomes in ovarian cancer [79], but their association with PAAD has not been documented.

Previous studies have shown that potential cancer biomarkers can be identified using DNA methylation profiles [80–82]. In this study, we could identify 12 DNA methylation of CpG probes affecting 14 genes as the predictive biomarkers of gemcitabine responses in PAAD patients by XGBoost-OMC model. The genes found to be associated with these CpG probes are presented in bracket: cg03208198 (COL18A1 and MIR6815), cg07239938 (ELANE), cg11528307 (C14orf80 and CRIP1), cg15200796 (TMEM191C), cg16049691 (AHRR), cg16379910 (B2M), cg17804635 (ZNF703), cg23854567 (PXN), cg24128434 (DNAH2), cg25653341 (PLOD3), cg27152190 (RP11-429P3.3), and ch.2.184672693R (not linked to any gene). The 2 CpG probes (cg115283072 and ch.2.184672693R) were commonly selected by other models that employed CpG profile, including CART-OMC, LGBM-OMC, and RF-OMC, with mMCC of 0.28, 0.18, and 0.18, respectively (Table S8). In addition, we also investigated the differential expression of gemcitabine response predictors and found that increased 4 mRNA expressions and DNA methylation of 11 CpG sites in pretreatment biopsies from gemcitabine-treated PAAD patients are correlated with better response to therapy, while increased DNA methylation of cg03208198 predicts resistance to therapy (Fig. 4). In fact, dysregulation of DNA methylation (hypo- or hypermethylation) is related to affected gene activation or associated with gene silencing including known tumor suppressor genes and loss of gene functions in cancer [83]. Some of the genes corresponding to the predictive CpG probes have been previously reported to be associated with the prognosis and clinical outcomes in various cancers. The high expression of COL18A1 in PC [84], gastric cancer [85], and esophageal squamous cell carcinoma [86] showed poor clinical outcomes. Down-regulation of CRIP1 has also been identified in invasive PC [87]. ZNF703 is an oncogenic transcriptional regulator involved in cell adhesion, movement, and proliferation [88]. Overexpression of ZNF703 is linked to tamoxifen resistance [89]. From this subset of predictive genes, we carried out PPI and pathway analyses to further support follow-up mechanistic studies. Here, we found that the genes corresponding to predictive CpG probes were significantly enriched in a number of cancer-associated pathways and drug resistance from GO and KEGG pathway database (Fig. S5 and Tables S4 and S5).

We next analyzed the survival difference observed between predicted responders and nonresponders by using the best models among gemcitabine-treated PAAD patients. The Kaplan–Meier survival analysis shows that the median OS and PFS were not significantly different between the actual and the predicted responders and nonresponders from the best models. Both OS and PFS were increased in responders compared to nonresponders (Figs. 7 and 8). The univariate analysis suggests that gemcitabine response predictors could be used as independent features for survival prediction of PAAD patients receiving gemcitabine (Fig. S7). Some studies have reported these genes as predictors of PAAD survival outcomes [68,90]. Note that building a better predictor of PAAD survival would require considering all PAAD patients regardless of the administered drug, which is out of the scope of this study.

Additionally, merging features did not improve the model performances (Fig. 9A). Clinical features alone were barely predictive, and combining them with predictive mRNA profile did not improve the model performance. Furthermore, there is no improvement by integrating 2 most predictive molecular profiles. In addition, loss in predictive accuracy was observed when combining all molecular profiles and clinical features. These suggest that RF-OMC was not able to capture the hidden informative features when the dimensionality dataset exceeded 500,000 features.

Furthermore, several studies have reported hENT1 expression as a gemcitabine response biomarker in PC [16,17], but the mMCC across five 10-fold CV runs was barely predictive (mMCC ~ 0), while the prediction based on 4 predictive mRNAs (mMCC of 0.44) and 12 predictive CpGs (mMCC of 0.32) achieved a significantly higher performance (Fig. 9B). This could suggest the attractiveness of the identified biosignatures. However, the improvement of these ML models over the hENT1marker will have to be more accurately quantified in a prospective clinical trial.

Although the best models, based on mRNAs and CpG profiles, have predictive and robust performances, there are several limitations to be considered. First, this is a retrospective study using publicly available data from TCGA-PAAD project. While the number of patients is large for a cancer-specific drug response study (diagnosis or prognosis datasets are not restricted to a single administered drug and hence are larger), a larger cohort would further improve model performance. Indeed, as shown in Fig. 6, the more data used to train the model, the more accurate the model. Second, an independent validation cohort would further test the generalizability of the best model, i.e., whether this is able to stratify patients with different characteristics or technical validations (e.g., sample preparation, sequencing procedure, and bioinformatics pipeline). With that said, this is not critical here as it is when using clinical trial data, as TCGA data come from clinical practice in different hospitals. Third, the effect of drug combinations could be further evaluated along with its individual effect, as a few patients were given other drugs in combination with gemcitabine.

## Conclusions

In summary, this systematic ML analysis revealed the discovery of predictive, reproducible, and robust gene expression and DNA methylation computational models for gemcitabine-treated PAAD patients. These models can be employed as response signatures to guide clinicians to stratify PAAD patients who will respond to gemcitabine in order to propose other suitable drugs without delay and avoid side effects to patients who are unlikely to respond. In addition, these signatures could be a starting point to explore the gemcitabine resistance mechanism.

## Data Availability

The datasets supporting the results of this article are open access data downloaded from the TCGA-PAAD project within the GDC resource (https://portal.gdc.cancer.gov/). The python code and processed mRNA(FPKM) dataset are provided in order to build the best model to predict and evaluate gemcitabine response in PAAD patients and facilitate their application to other cohorts of mRNA-profiled PAAD patients: https://github.com/chayanitpiy/gemcitabine_PAAD

## References

[1] Sung H, Ferlay J, Siegel RL, Laversanne M, Soerjomataram I, Jemal A, Bray F. Global cancer statistics 2020: GLOBOCAN estimates of incidence and mortality worldwide for 36 cancers in 185 countries. CA Cancer J Clin. 2021;71(3):209–249. 33538338 10.3322/caac.21660

[2] Zhu H, Li T, Du Y, Li M. Pancreatic cancer: Challenges and opportunities. BMC Med. 2018;16(1):214. 30463539 10.1186/s12916-018-1215-3 PMC6249728

[3] Siegel RL, Miller KD, Fuchs HE, Jemal A. Cancer statistics, 2022. CA Cancer J Clin. 2022;72(1):7–33. 35020204 10.3322/caac.21708

[4] Jia Y, Xie J. Promising molecular mechanisms responsible for gemcitabine resistance in cancer. Genes Dis. 2015;2(4):299–306. 30258872 10.1016/j.gendis.2015.07.003 PMC6150077

[5] Zhang XW, Ma YX, Sun Y, Cao YB, Li Q, Xu CA. Gemcitabine in combination with a second cytotoxic agent in the first-line treatment of locally advanced or metastatic pancreatic cancer: A systematic review and meta-analysis. Target Oncol. 2017;12(3):309–321. 28353074 10.1007/s11523-017-0486-5

[6] Sarabi M, Mais L, Oussaid N, Desseigne F, Guibert P, De La Fouchardiere C. Use of gemcitabine as a second-line treatment following chemotherapy with folfirinox for metastatic pancreatic adenocarcinoma. Oncol Lett. 2017;13(6):4917–4924. 28599496 10.3892/ol.2017.6061 PMC5453056

[7] Nagourney RA, Flam M, Link J, Hager S, Blitzer J, Lyons W, Sommers BL, Evans S. Carboplatin plus gemcitabine repeating doublet therapy in recurrent breast cancer. Clin Breast Cancer. 2008;8(5):432–435. 18952557 10.3816/CBC.2008.n.052

[8] Pfisterer J, Plante M, Vergote I, du Bois A, Hirte H, LacaveUwe Wagner AJ, Stähle A, Stuart G, Kimmig R, Olbricht S, et al. Gemcitabine plus carboplatin compared with carboplatin in patients with platinum-sensitive recurrent ovarian cancer: An intergroup trial of the AGO-OVAR, the NCIC CTG, and the EORTC GCG. J Clin Oncol. 2006;24:(29)4699–4707. 16966687 10.1200/JCO.2006.06.0913

[9] von der Maase H, Hansen SW, Roberts JT, Dogliotti L, Oliver T, Moore MJ, Bodrogi I, Albers P, Knuth A, Lippert CM, et al. Gemcitabine and cisplatin versus methotrexate, vinblastine, doxorubicin, and cisplatin in advanced or metastatic bladder cancer: Results of a large, randomized, multinational, multicenter, phase III study. J Clin Oncol. 2000;18(17):3068–3077. 11001674 10.1200/JCO.2000.18.17.3068

[10] Sandler AB, Nemunaitis J, Denham C, von Pawel J, Cormier Y, Gatzemeier U, Mattson K, Manegold C, Palmer MC, Gregor A, et al. Phase III trial of gemcitabine plus cisplatin versus cisplatin alone in patients with locally advanced or metastatic non-small-cell lung cancer. J Clin Oncol. 2000;18(1):122–130. 10623702 10.1200/JCO.2000.18.1.122

[11] Amrutkar M, Gladhaug IP. Pancreatic cancer chemoresistance to gemcitabine. Cancers (Basel). 2017;9(11):157. 29144412 10.3390/cancers9110157 PMC5704175

[12] Berdis AJ. Inhibiting DNA polymerases as a therapeutic intervention against cancer. Front Mol Biosci. 2017;4:78. 29201867 10.3389/fmolb.2017.00078 PMC5696574

[13] Von Hoff DD, Ervin T, Arena FP, Gabriela Chiorean E, Infante J, Moore M, Seay T, Tjulandin SA, Ma WW, Saleh MN. Increased survival in pancreatic cancer with nab-paclitaxel plus gemcitabine. N Engl J Med. 2013;369(18):1691–1703. 24131140 10.1056/NEJMoa1304369 PMC4631139

[14] Bird NTE, Elmasry M, Jones R, Psarelli E, Dodd J, Malik H, Greenhalf W, Kitteringham N, Ghaneh P, Neoptolemos JP, et al. Immunohistochemical hENT1 expression as a prognostic biomarker in patients with resected pancreatic ductal adenocarcinoma undergoing adjuvant gemcitabine-based chemotherapy. Br J Surg. 2017;104(4):328–336. 28199010 10.1002/bjs.10482

[15] Wei CH, Gorgan TR, Elashoff DA, Hines OJ, Farrell JJ, Donahue TR. A meta-analysis of gemcitabine biomarkers in patients with pancreatico-biliary cancers. Pancreas. 2013;42(8):1303–1310. 24152955 10.1097/MPA.0b013e3182a23ae4 PMC3813307

[16] Spratlin J, Sangha R, Glubrecht D, Dabbagh L, Young JD, Dumontet C, Cass C, Lai R, Mackey JR. The absence of human equilibrative nucleoside transporter 1 is associated with reduced survival in patients with gemcitabine-treated pancreas adenocarcinoma. Clin Cancer Res. 2004;10(20):6956–6961. 15501974 10.1158/1078-0432.CCR-04-0224

[17] Farrell JJ, Elsaleh H, Garcia M, Lai R, Ammar A, Regine WF, Abrams R, Benson AB, Macdonald J, Cass CE, et al. Human equilibrative nucleoside transporter 1 levels predict response to gemcitabine in patients with pancreatic cancer. Gastroenterology. 2009;136(1):187–195. 18992248 10.1053/j.gastro.2008.09.067

[18] Giovannetti E, Tacca MD, Mey V, Funel N, Nannizzi S, Ricci S, Orlandini C, Boggi U, Campani D, Chiaro MD, et al. Transcription analysis of human equilibrative nucleoside transporter-1 predicts survival in pancreas cancer patients treated with gemcitabine. Cancer Res. 2006;66(7):3928–3935. 16585222 10.1158/0008-5472.CAN-05-4203

[19] Wang W, Yu X, Li H, Yang C, Jin C, Huang X. hENT1’s role in adjuvant intra-arterial gemcitabine-based chemotherapy for resectable pancreatic cancer patients. BMC Gastroenterol. 2023;23(1):35. 36755224 10.1186/s12876-023-02666-x PMC9909848

[20] Kawada N, Uehara H, Katayama K, Nakamura S, Takahashi H, Ohigashi H, Ishikawa O, Nagata S, Tomita Y. Human equilibrative nucleoside transporter 1 level does not predict prognosis in pancreatic cancer patients treated with neoadjuvant chemoradiation including gemcitabine. J Hepatobiliary Pancreat Sci. 2012;19(6):717–722. 22426593 10.1007/s00534-012-0514-x

[21] Yabushita Y, Mori R, Taniguchi K, Matsuyama R, Kumamoto T, Sakamaki K, Kubota K, Endo I. Combined analyses of hENT1, TS, and DPD predict outcomes of borderline-resectable pancreatic cancer. Anticancer Res. 2017;37(5):2465–2476. 28476815 10.21873/anticanres.11587

[22] Liu X, Xiao C, Yue K, Chen M, Zhou H, Yan X. Identification of multi-omics biomarkers and construction of the novel prognostic model for hepatocellular carcinoma. Sci Rep. 2022;12(1):12084. 35840618 10.1038/s41598-022-16341-w PMC9287549

[23] Kong L, Liu P, Zheng M, Xue B, Liang K, Tan X. Multi-omics analysis based on integrated genomics, epigenomics and transcriptomics in pancreatic cancer. Epigenomics. 2020;12(6):507–524. 32048534 10.2217/epi-2019-0374

[24] Guinney J, Wang T, Laajala TD, Winner KK, Bare JC, Neto EC, Khan SA, Peddinti G, Airola A, Pahikkala T, et al. Prediction of overall survival for patients with metastatic castration-resistant prostate cancer: Development of a prognostic model through a crowdsourced challenge with open clinical trial data. Lancet Oncol. 2017;18(1):132–142. 27864015 10.1016/S1470-2045(16)30560-5 PMC5217180

[25] Seyednasrollah F, Koestler DC, Wang T, Piccolo SR, Vega R, Greiner R, Fuchs C, Gofer E, Kumar L, Wolfinger RD, et al. Prostate Cancer DREAM Challenge Community. A DREAM challenge to build prediction models for short-term discontinuation of docetaxel in metastatic castration-resistant prostate cancer. JCO Clin Cancer Inform. 2017;1:1–15. 10.1200/CCI.17.00018 PMC6874023 30657384

[26] Ghislat G, Cheema AS, Baudoin E, Verthuy C, Ballester PJ, Crozat K, Attaf N, Dong C, Milpied P, Malissen B, et al. NF-κB–dependent IRF1 activation programs cDC1 dendritic cells to drive antitumor immunity. Sci Immunol. 2021;6(61): Article eabg3570. 34244313 10.1126/sciimmunol.abg3570

[27] Menden MP, Wang D, Mason MJ, Szalai B, Bulusu KC, Guan Y, Yu T, Kang J, Jeon M, Wolfinger R, et al. Community assessment to advance computational prediction of cancer drug combinations in a pharmacogenomic screen. Nat Commun. 2019;10(1): Article 2674. 31209238 10.1038/s41467-019-09799-2 PMC6572829

[28] Piyawajanusorn C, Nguyen LC, Ghislat G, Ballester PJ. A gentle introduction to understanding preclinical data for cancer pharmaco-omic modeling. Brief Bioinform. 2021;22(6): Article bbab312. 34368843 10.1093/bib/bbab312

[29] Dang CC, Peón A, Ballester PJ. Unearthing new genomic markers of drug response by improved measurement of discriminative power. BMC Med Genomics. 2018;11(1):10. 29409485 10.1186/s12920-018-0336-z PMC5801688

[30] Garnett MJ, Edelman EJ, Heidorn SJ, Greenman CD, Dastur A, Lau KW, Greninger P, Thompson IR, Luo X, Soares J, et al. Systematic identification of genomic markers of drug sensitivity in cancer cells. Nature. 2012;483(7391):570–575. 22460902 10.1038/nature11005 PMC3349233

[31] Frejno M, Chiozzi RZ, Wilhelm M, Koch H, Zheng R, Klaeger S, Ruprecht B, Meng C, Kramer K, Jarzab A, et al. Pharmacoproteomic characterisation of human colon and rectal cancer. Mol Syst Biol. 2017;13(11):951. 29101300 10.15252/msb.20177701 PMC5731344

[32] Chibon F. Cancer gene expression signatures—The rise and fall? Eur J Cancer. 2013;49(8):2000–2009. 23498875 10.1016/j.ejca.2013.02.021

[33] Liu K, Geng Y, Wang L, Xu H, Zou M, Li Y, Zhao Z, Chen T, Xu F, Sun L, et al. Systematic exploration of the underlying mechanism of gemcitabine resistance in pancreatic adenocarcinoma. Mol Oncol. 2022;16(16):3034–3051. 35810469 10.1002/1878-0261.13279 PMC9394232

[34] Ballester PJ, Stevens R, Haibe-Kains B, Huang RS, Aittokallio T. Artificial intelligence for drug response prediction in disease models. Brief Bioinform. 2022;23(1): Article bbab450. 34655289 10.1093/bib/bbab450

[35] Malik V, Kalakoti Y, Sundar D. Deep learning assisted multi-omics integration for survival and drug-response prediction in breast cancer. BMC Genomics. 2021;22(1): Article 214. 33761889 10.1186/s12864-021-07524-2 PMC7992339

[36] Yu L, Zhou D, Gao L, Zha Y. Prediction of drug response in multilayer networks based on fusion of multiomics data. Methods. 2021;192:85–92. 32798653 10.1016/j.ymeth.2020.08.006

[37] Dorman SN, Baranova K, Knoll JHM, Urquhart BL, Mariani G, Carcangiu ML, Rogan PK. Genomic signatures for paclitaxel and gemcitabine resistance in breast cancer derived by machine learning. Mol Oncol. 2016;10(1):85–100. 26372358 10.1016/j.molonc.2015.07.006 PMC5528934

[38] Menden MP, Iorio F, Garnett M, Dermott UM, Benes CH, Ballester PJ, Saez-Rodriguez J. Machine learning prediction of cancer cell sensitivity to drugs based on genomic and chemical properties. PLOS ONE. 2013;8(4): Article e61318. 23646105 10.1371/journal.pone.0061318 PMC3640019

[39] Naulaerts S, Dang CC, Ballester PJ. Precision and recall oncology: Combining multiple gene mutations for improved identification of drug-sensitive tumours. Oncotarget. 2017;8(57):97025–97040. 29228590 10.18632/oncotarget.20923 PMC5722542

[40] Nguyen L, Dang CC, Ballester PJ. Systematic assessment of multi-gene predictors of pan-cancer cell line sensitivity to drugs exploiting gene expression data. F1000Res. 2016;5. 10.12688/f1000research.10529.1 PMC5310525 28299173

[41] Naulaerts S, Menden MP, Ballester PJ. Concise polygenic models for cancer-specific identification of drug-sensitive tumors from their multi-omics profiles. Biomolecules. 2020;10(6): Article 963. 32604779 10.3390/biom10060963 PMC7356608

[42] Ammad-Ud-Din M, Khan SA, Malani D, Murumägi A, Kallioniemi O, Aittokallio T, Kaski S. Drug response prediction by inferring pathway-response associations with kernelized Bayesian matrix factorization. Bioinformatics. 2016;32(17):i455–i463. 27587662 10.1093/bioinformatics/btw433

[43] Ballester PJ, Carmona J. Artificial intelligence for the next generation of precision oncology. NPJ Precis Oncol. 2021;5(1): Article 79. 10.1038/s41698-021-00216-w PMC8373978 34408248

[44] Bomane A, Gonçalves A, Ballester PJ. Paclitaxel response can be predicted with interpretable multi-variate classifiers exploiting DNA-methylation and miRNA data. Front Genet. 2019;10: Article 1041. 31708973 10.3389/fgene.2019.01041 PMC6823251

[45] Ogunleye AZ, Piyawajanusorn C, Gonçalves A, Ghislat G, Ballester PJ. Interpretable machine learning models to predict the resistance of breast cancer patients to doxorubicin from their microRNA profiles. Adv Sci (Weinh). 2022;9(24): Article e2201501. 35785523 10.1002/advs.202201501 PMC9403644

[46] Xu Y, Dong Q, Li F, Xu Y, Hu C, Wang J, Shang D, Zheng X, Yang H, Zhang C, et al. Identifying subpathway signatures for individualized anticancer drug response by integrating multi-omics data. J Transl Med. 2019;17(1): Article 227. 31387579 10.1186/s12967-019-2010-4 PMC6685260

[47] Krishna V, Nimgaonkar V, Tiu E, Krishna V, Bhambhvani H, Cook S, Miller D, Vrabac D, Joshi A, Singhi AD, et al. Gemcitabine response prediction in the adjuvant treatment of resected pancreatic ductal adenocarcinoma using an AI histopathology platform. J Clin Oncol. 2022;40(16):e16295–e16295.

[48] Clayton EA, Pujol TA, McDonald JF, Qiu P. Leveraging TCGA gene expression data to build predictive models for cancer drug response. BMC Bioinformatics. 2020;21(Suppl 14):364. 32998700 10.1186/s12859-020-03690-4 PMC7526215

[49] Foersch S, Glasner C, Woerl A-C, Eckstein M, Wagner D-C, Schulz S, Kellers F, Fernandez A, Tserea K, Kloth M, et al. Multistain deep learning for prediction of prognosis and therapy response in colorectal cancer. Nat Med. 2023;(2):430–439. 36624314 10.1038/s41591-022-02134-1

[50] Nguyen LC, Naulaerts S, Bruna A, Ghislat G, Ballester PJ. Predicting cancer drug response in vivo by learning an optimal feature selection of tumour molecular profiles. Biomedicines. 2021;9(10):1319. 34680436 10.3390/biomedicines9101319 PMC8533095

[51] Cancer Genome Atlas Research Network, Weinstein JN, Collisson EA, Mills GB, Shaw KRM, Ozenberger BA, Ellrott K, Shmulevich I, Sander C, Stuart JM. The Cancer Genome Atlas Pan-Cancer analysis project. Nat Genet. 2013;45(10):1113–1120. 24071849 10.1038/ng.2764 PMC3919969

[52] Kleeff J, Korc M, Apte M, Vecchia CL, Johnson CD, Biankin AV, Neale RE, Tempero M, Tuveson DA, Hruban RH, et al. Pancreatic cancer. Nat Rev Dis Primers. 2016;2:–16022. 10.1038/nrdp.2016.22 27158978

[53] Szklarczyk D, Gable AL, Nastou KC, Lyon D, Kirsch R, Pyysalo S, Doncheva NT, Legeay M, Fang T, Bork P, et al. The STRING database in 2021: Customizable protein-protein networks, and functional characterization of user-uploaded gene/measurement sets. Nucleic Acids Res. 2021;49(D1):D605–D612. 33237311 10.1093/nar/gkaa1074 PMC7779004

[54] Jiao X, Sherman BT, Huang DW, Stephens R, Baseler MW, Lane HC, Lempicki RA. DAVID-WS: A stateful web service to facilitate gene/protein list analysis. Bioinformatics. 2012;28(13):1805–1806. 22543366 10.1093/bioinformatics/bts251 PMC3381967

[55] Bu D, Luo H, Huo P, Wang Z, Zhang S, He Z, Wu Y, Zhao L, Liu J, Guo J, et al. KOBAS-i: Intelligent prioritization and exploratory visualization of biological functions for gene enrichment analysis. Nucleic Acids Res. 2021;49(W1):W317–W325. 34086934 10.1093/nar/gkab447 PMC8265193

[56] Cooper GM. The *development and causes of cancer*. Sunderland (MA): Sinauer Associates; 2000.

[57] Wittekind C, Neid M. Cancer invasion and metastasis. Oncology. 2005;69(Suppl 1):14–16. 16210871 10.1159/000086626

[58] Xiong G-F, Xu R. Function of cancer cell-derived extracellular matrix in tumor progression. J Cancer Metastasis Treat. 2016;2(9):357–364.

[59] Stewart BW. Mechanisms of carcinogenesis: From initiation and promotion to the hallmarks. In: Baan RA, Stewart BW, Straif K, editors. Tumour site concordance and mechanisms of carcinogenesis. Lyon (France): International Agency for Research on Cancer; 2019. 33979080

[60] Sever R, Brugge JS. Signal transduction in cancer. Cold Spring Harb Perspect Med. 2015;5(4):a006098. 25833940 10.1101/cshperspect.a006098 PMC4382731

[61] Andl CD. The misregulation of cell adhesion components during tumorigenesis: Overview and commentary. J Oncol. 2010;2010: Article 174175. 10.1155/2010/174715 PMC2952821 20953359

[62] Linzer N, Trumbull A, Nar R, Gibbons MD, Yu DT, Strouboulis J, Bungert J. Regulation of RNA polymerase II transcription initiation and elongation by transcription factor TFII-I. Front Mol Biosci. 2021;8: Article 681550. 34055891 10.3389/fmolb.2021.681550 PMC8155576

[63] Doehmer J, Goeptar AR, Vermeulen NP. Cytochromes P450 and drug resistance. Cytotechnology. 1993;12(1–3):357–366. 7764457 10.1007/BF00744673

[64] Mpakali A, Stratikos E. The role of antigen processing and presentation in cancer and the efficacy of immune checkpoint inhibitor immunotherapy. Cancers (Basel). 2021;13(1):134. 33406696 10.3390/cancers13010134 PMC7796214

[65] García-Manteiga J, Molina-Arcas M, Casado FJ, Mazo A, Pastor-Anglada M. Nucleoside transporter profiles in human pancreatic cancer cells: Role of hCNT1 in 2,2-difluorodeoxycytidine-induced cytotoxicity. Clin Cancer Res. 2003;**9**(13):5000–5008. 14581375

[66] Nakano Y, Tanno S, Koizumi K, Nishikawa T, Nakamura K, Minoguchi M, Izawa T, Mizukami Y, Okumura T, Kohgo Y. Gemcitabine chemoresistance and molecular markers associated with gemcitabine transport and metabolism in human pancreatic cancer cells. Br J Cancer. 2007;96(3):457–463. 17224927 10.1038/sj.bjc.6603559 PMC2360025

[67] Voutsadakis IA. Molecular predictors of gemcitabine response in pancreatic cancer. World J Gastrointest Oncol. 2011;3(11):153–164. 22110842 10.4251/wjgo.v3.i11.153 PMC3220724

[68] Wei X, Zhou X, Zhao Y, He Y, Weng Z, Xu C. A 14-gene gemcitabine resistance gene signature is significantly associated with the prognosis of pancreatic cancer patients. Sci Rep. 2021;11(1):6087. 33731794 10.1038/s41598-021-85680-x PMC7969955

[69] Nicolle R, Gayet O, Duconseil P, Vanbrugghe C, Roques J, Bigonnet M, Blum Y, Elarouci N, Armenoult L, Ayadi M, et al. A transcriptomic signature to predict adjuvant gemcitabine sensitivity in pancreatic adenocarcinoma. Ann Oncol. 2021;32(2):250–260. 33188873 10.1016/j.annonc.2020.10.601

[70] Mehrmohamadi M, Jeong SH, Locasale JW. Molecular features that predict the response to antimetabolite chemotherapies. Cancer Metab. 2017;5:8. 29026541 10.1186/s40170-017-0170-3 PMC5627437

[71] Schuh A, Dreau H, Knight SJL, Ridout K, Mizani T, Vavoulis D, Colling R, Antoniou P, Kvikstad EM, Pentony MM, et al. Clinically actionable mutation profiles in patients with cancer identified by whole-genome sequencing. Cold Spring Harb Mol Case Stud. 2018;4(2): Article a002279. 29610388 10.1101/mcs.a002279 PMC5880257

[72] Tanaka M, Javle M, Dong X, Eng C, Abbruzzese JL, Li D. Gemcitabine metabolic and transporter gene polymorphisms are associated with drug toxicity and efficacy in patients with locally advanced pancreatic cancer. Cancer. 2010;116(22):5325–5335. 20665488 10.1002/cncr.25282 PMC2966859

[73] Rodríguez-Antona C, Taron M. Pharmacogenomic biomarkers for personalized cancer treatment. J Intern Med. 2015;277(2):201–217. 25338550 10.1111/joim.12321

[74] Huang M, Shen A, Ding J, Geng M. Molecularly targeted cancer therapy: Some lessons from the past decade. Trends Pharmacol Sci. 2014;35(1):41–50. 24361003 10.1016/j.tips.2013.11.004

[75] He D-X, Gu F, Gao F, Hao J-J, Gong D, Gu X-T, Mao A-Q, Jin J, Fu L, Ma X. Genome-wide profiles of methylation, microRNAs, and gene expression in chemoresistant breast cancer. Sci Rep. 2016;6: Article 24706. 27094684 10.1038/srep24706 PMC4837395

[76] Costello JC, Heiser LM, Georgii E, Gönen M, Menden MP, Wang NJ, Bansal M, Ammad-ud-din M, Hintsanen P, Khan SA, et al. A community effort to assess and improve drug sensitivity prediction algorithms. Nat Biotechnol. 2014;32(12):1202–1212. 24880487 10.1038/nbt.2877 PMC4547623

[77] Tsamardinos I, Charonyktakis P, Papoutsoglou G, Borboudakis G, Lakiotaki K, Zenklusen JC, Juhl H, Chatzaki E, Lagani V. Just add data: Automated predictive modeling for knowledge discovery and feature selection. NPJ Precis Oncol. 2022;6(1):38. 35710826 10.1038/s41698-022-00274-8 PMC9203777

[78] Tan L, Mai D, Zhang B, Jiang X, Zhang J, Bai R, Ye Y, Li M, Pan L, Su J, et al. PIWI-interacting RNA-36712 restrains breast cancer progression and chemoresistance by interaction with SEPW1 pseudogene SEPW1P RNA. Mol Cancer. 2019;18(1):9. 30636640 10.1186/s12943-019-0940-3 PMC6330501

[79] Johnatty SE, Tyrer JP, Kar S, Beesley J, Lu Y, Gao B, Fasching PA, Hein A, Ekici AB, Beckmann MW, et al. Genome-wide analysis identifies novel loci associated with ovarian cancer outcomes: Findings from the Ovarian Cancer Association Consortium. Clin Cancer Res. 2015;21(23):5264–5276. 26152742 10.1158/1078-0432.CCR-15-0632 PMC4624261

[80] Stirzaker C, Taberlay PC, Statham AL, Clark SJ. Mining cancer methylomes: Prospects and challenges. Trends Genet. 2014;30(2):75–84. 24368016 10.1016/j.tig.2013.11.004

[81] Mikeska T, Craig JM. DNA methylation biomarkers: Cancer and beyond. Genes (Basel). 2014;5(3):821–864. 25229548 10.3390/genes5030821 PMC4198933

[82] Levenson VV. DNA methylation as a universal biomarker. Expert Rev Mol Diagn. 2010;10(4):481–488. 20465502 10.1586/erm.10.17 PMC2933138

[83] Ehrlich M. DNA methylation in cancer: Too much, but also too little. Oncogene. 2002;21(35):5400–5413. 12154403 10.1038/sj.onc.1205651

[84] Nakajima K, Ino Y, Naito C, Nara S, Shimasaki M, Ishimoto U, Iwasaki T, Doi N, Esaki M, Kishi Y, et al. Neoadjuvant therapy alters the collagen architecture of pancreatic cancer tissue via Ephrin-A5. Br J Cancer. 2022;126(4):628–639. 34824448 10.1038/s41416-021-01639-9 PMC8854423

[85] Weng K, Huang Y, Deng H, Wang R, Luo S, Wu H, Chen J, Long M, Hao W. Collagen family genes and related genes might be associated with prognosis of patients with gastric cancer: An integrated bioinformatics analysis and experimental validation. Transl Cancer Res. 2020;9(10):6246–6262. 35117235 10.21037/tcr-20-1726 PMC8797647

[86] Li J, Wang X, Zheng K, Liu Y, Li J, Wang S, Liu K, Song X, Li N, Xie S, et al. The clinical significance of collagen family gene expression in esophageal squamous cell carcinoma. PeerJ. 2019;7:e7705. 31598423 10.7717/peerj.7705 PMC6779144

[87] Terris B, Blaveri E, Crnogorac-Jurcevic T, Jones M, Missiaglia E, Ruszniewski P, Sauvanet A, Lemoine NR. Characterization of gene expression profiles in intraductal papillary-mucinous tumors of the pancreas. Am J Pathol. 2002;160(5):1745–1754. 12000726 10.1016/S0002-9440(10)61121-2 PMC1850868

[88] Yang F, Teves SS, Kemp CJ, Henikoff S. Doxorubicin, DNA torsion, and chromatin dynamics. Biochim Biophys Acta. 2014;1845(1):84–89. 24361676 10.1016/j.bbcan.2013.12.002 PMC3927826

[89] Zhang X, Mu X, Huang O, Xie Z, Jiang M, Geng M, Shen K. Luminal breast cancer cell lines overexpressing ZNF703 are resistant to tamoxifen through activation of Akt/mTOR signaling. PLOS ONE. 2013;8(8):e72053. 23991038 10.1371/journal.pone.0072053 PMC3753350

[90] Wu M, Li X, Zhang T, Liu Z, Zhao Y. Identification of a nine-gene signature and establishment of a prognostic nomogram predicting overall survival of pancreatic cancer. Front Oncol. 2019;9:9996. 10.3389/fonc.2019.00996 PMC6776930 31612115

