# Supplementary material for: Large-Scale Machine Learning Analysis Reveals DNA Methylation and Gene Expression Response Signatures for Gemcitabine-Treated Pancreatic Cancer
Source: Health Data Sci. 2024 Jan 8;4:0108. doi: 10.34133/hds.0108 (PMC10904073; doi:10.34133/hds.0108)
Supplement: Supplementary 1 — Figs. S1 to 10 Tables S1 to S8 [file hds.0108.f1.docx]

Supplementary Materials

Supplementary figures


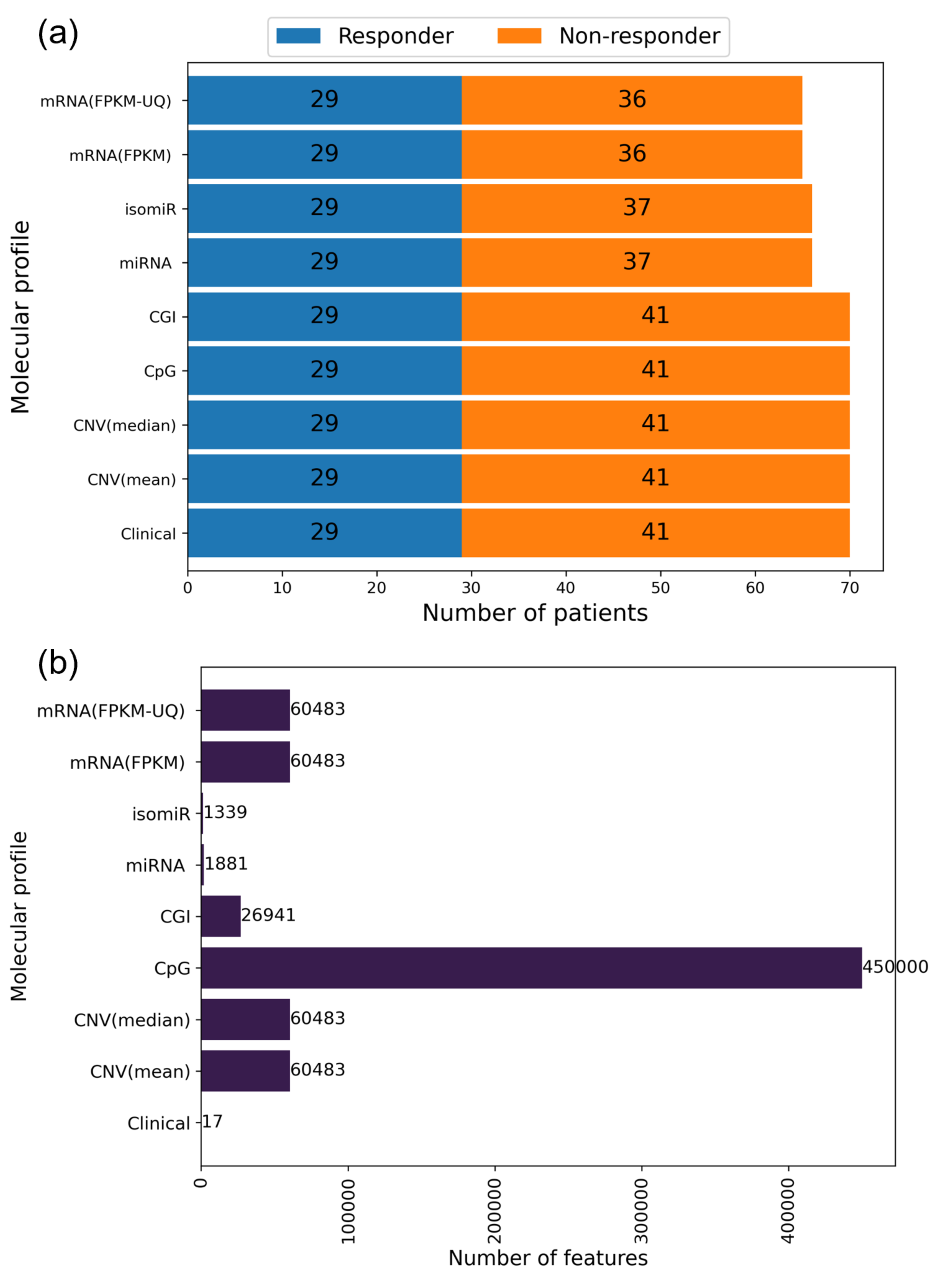


**Figure S1.** Bar charts presenting the patients distribution and the number of features available in each molecular profile. (a) The number of PAAD patients receiving gemcitabine in clinical and a given molecular profile dataset, where the majority of patients are non-responders. (b) the number of features available in each molecular profile, from 1,881 miRNAs to 450,000 CpG probes (DNA methylation). These highlight the variability and complexity of the molecular profiles used in this study to build predictive models for gemcitabine response in PAAD patients.

**
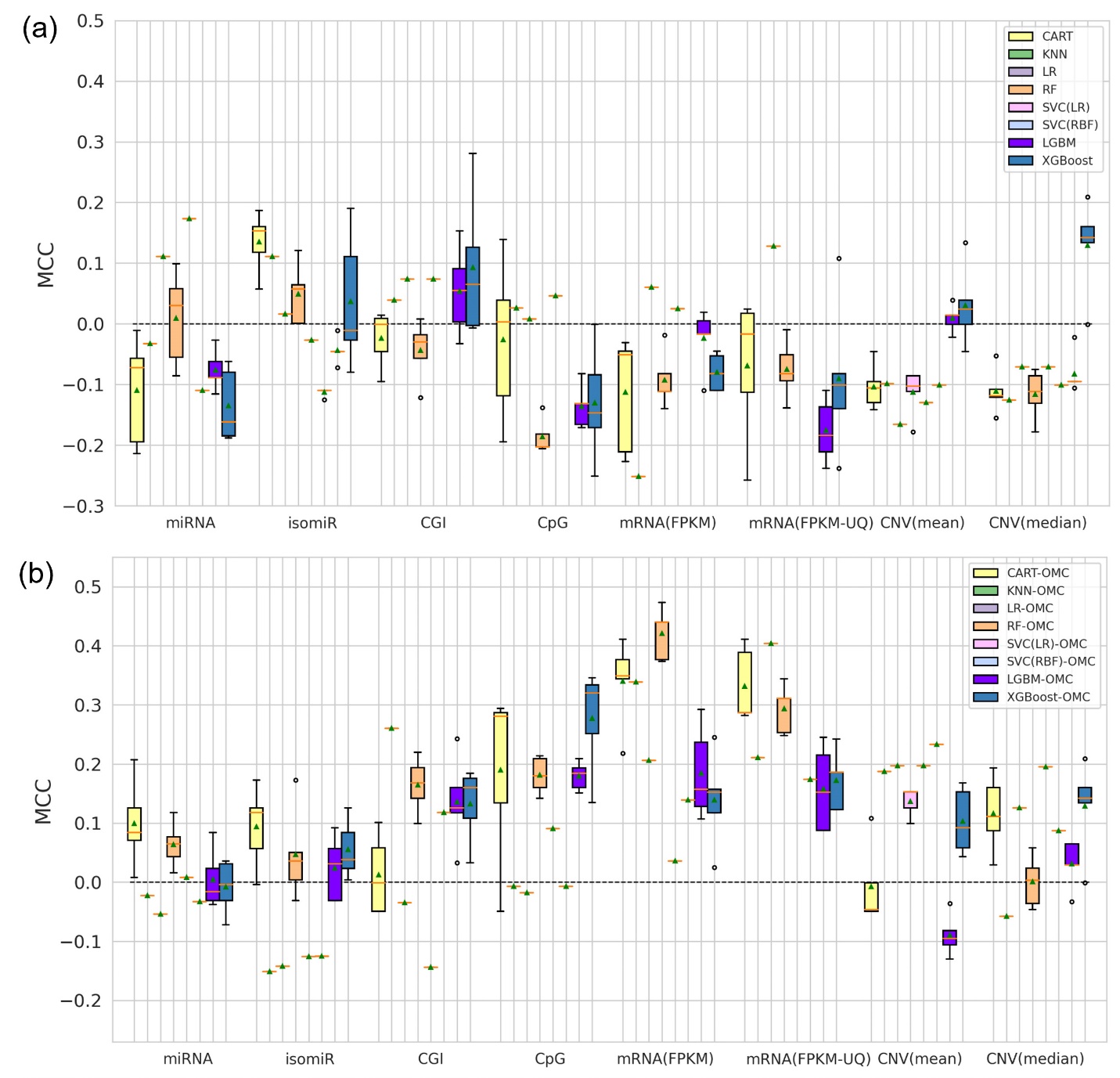
**

**Figure S2 (Related to Figure 2). Boxplots showing the 5 MCCs for each of the 128 ML models across five 10-fold CV runs.** The horizontal axis presents the 8 considered molecular profiles, while each boxplot shows the 5 MCCs, one for each 10-fold CV repetition on a given profile-algorithm pair. 16 binary classification algorithms were used: (a) 8 algorithms using all available features from the profile and (b) 8 algorithms integrated with OMC feature selection considering only the subset of informative features during the model training (the suffix “OMC” was added to the algorithm name). 10-fold CV with 5 repetitions, each repetition with different random seed, were carried out to evaluate the model performance. The out-of-sample prediction obtained from each CV were merged to calculate the evaluation metrics, thereby generating five MCCs (one MCC for each random seed) for each algorithm molecular profile pairs. Models that gave undefined MCC are not represented in the boxplots.


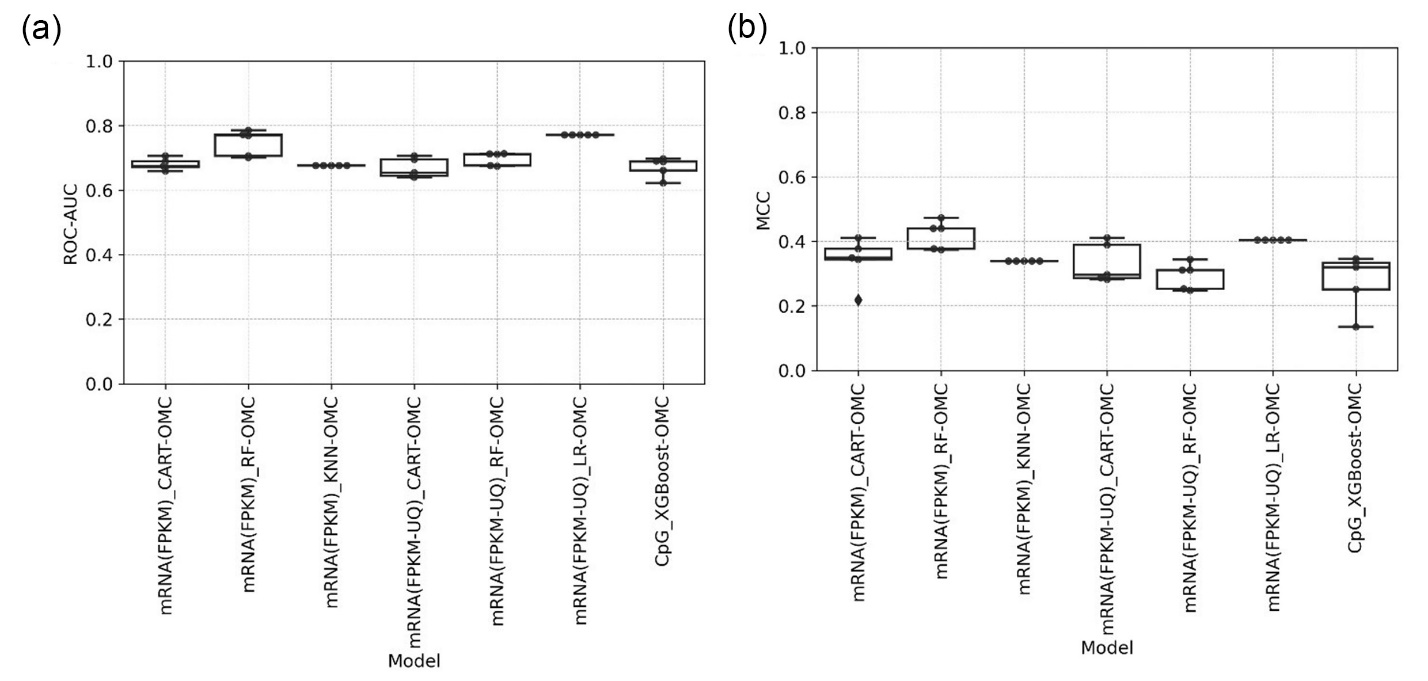


**Figure S3. The box plot presenting the model performance of the 7 most predictive models with mMCC at least 0.3**. (a) The ROC-AUC and (b) MCC obtained from five 10-fold CV runs, each runs with different random seeds. It can be observed that the models with good MCC also obtained good ROC-AUC. ROC-AUC describes how good the model is at predicting the positive class when the actual outcome is positive. While MCC is a more reliable which produces a high score only if the prediction obtained good results in all the 4 confusion matrix categories (true positives, false negatives, true negatives, and false positives). Therefore, we considered MCC as the main evaluation metric in binary classification problem.


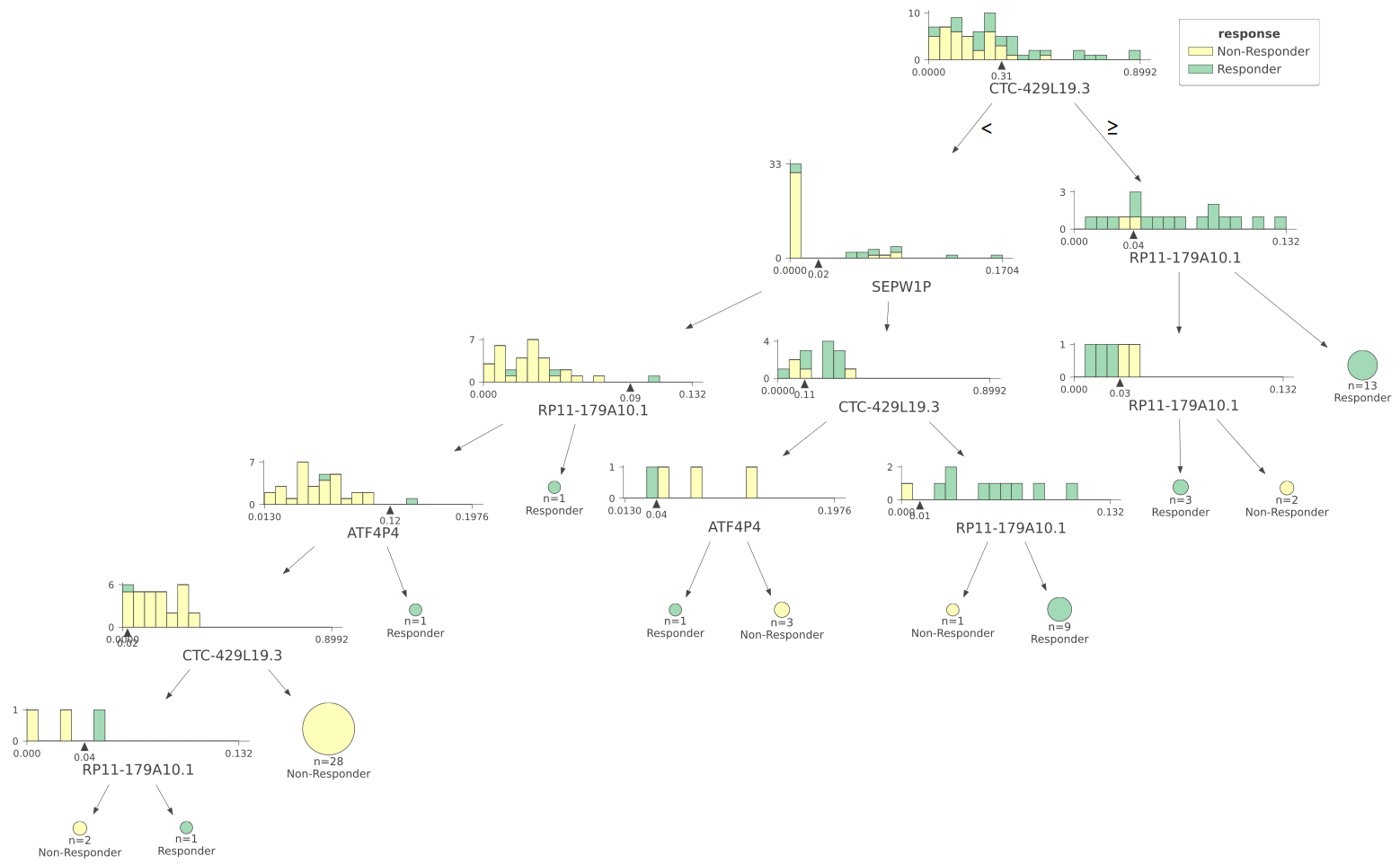


**Figure S4. Binary classification tree prediction of CART-OMC model with 4 predictive mRNAs on PAAD patients receiving gemcitabine.** The tree nodes are split the patients in to responders and non-responders based on the selected features The histogram at each tree node represents the distribution of patients at the selected features. The value at the best split for each feature is indicated by the triangle under each histogram along with feature name. Each node has two leaves; the patients with a feature value greater than or equal to the best split were split to the right, and vice versa. Terminal leaves are displayed as pie charts. The proportions of non-responders and responders are colored yellow and green, respectively. This decision tree reveals 4 mRNAs (CTC-429L19.3, SEPW1P, RP11-179A10.1 and ATF4P4) that could be used to predict the sensitivity and resistance of PAAD tumors to gemcitabine treatments.


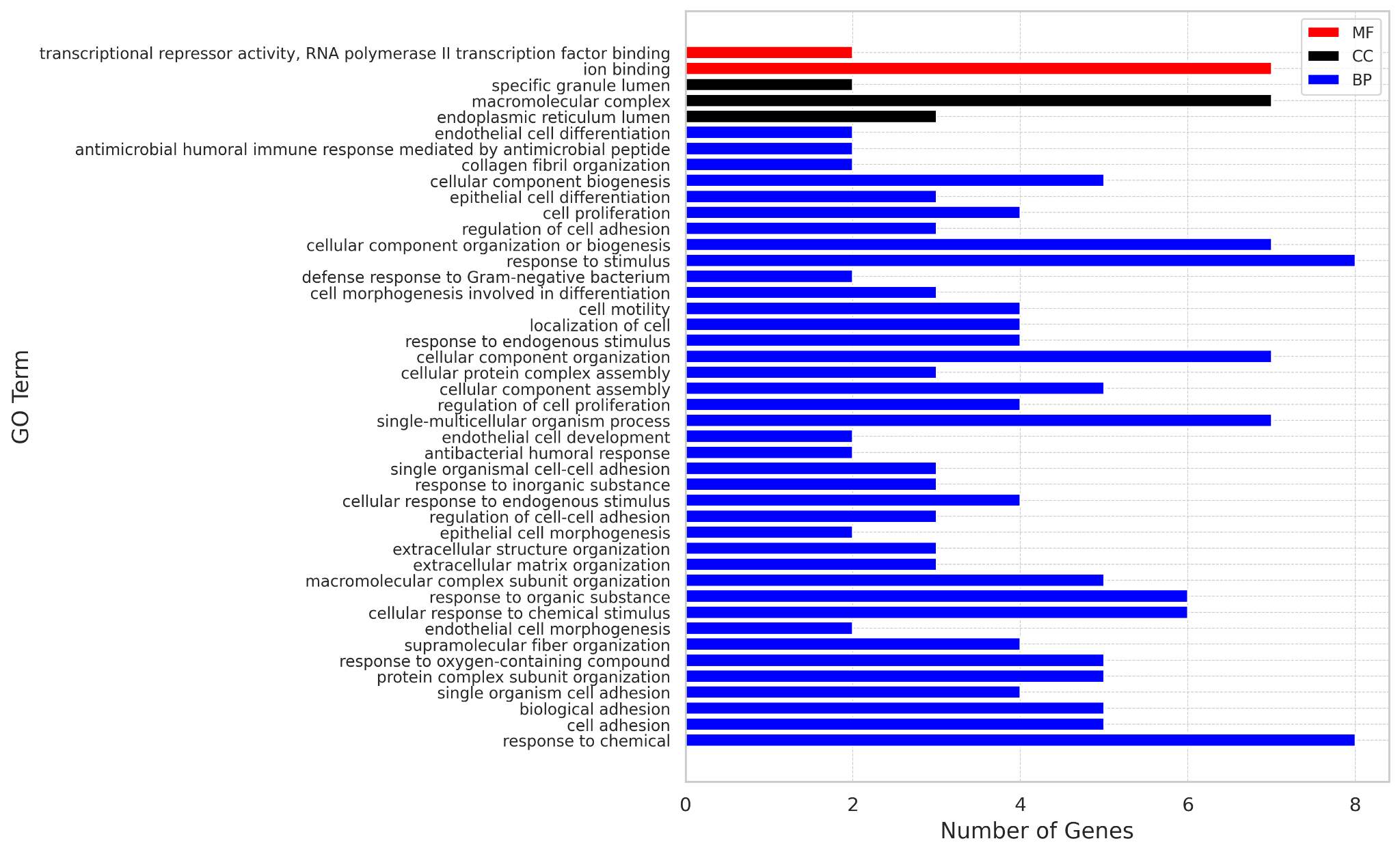


**Figure S5. GO enrichment analysis of the genes associated with the 12 predictive CpG probes for predicting gemcitabine response in PAAD patients.** The GO enrichment analysis was performed using DAVID web server. The significantly (p-value < 0.05) enriched GO terms in biological process (BP), molecular function (MF), and cellular component (CC) are summarized in the bar chart and presented on the y-axis, while the bars present the number of genes enriched in each GO terms. We could identify 43 significantly enriched GO pathways across BP, CC, and MF terms. The biological process pathways were primarily cancer-related, including cell differentiation, proliferation, adhesion, and motility, and provided insight into the underlying biological mechanisms driving cancer development. We also found that dysregulation of 8 genes predominantly enriched in response to chemical or stimulus pathways which could promote carcinogenesis and drug resistance.


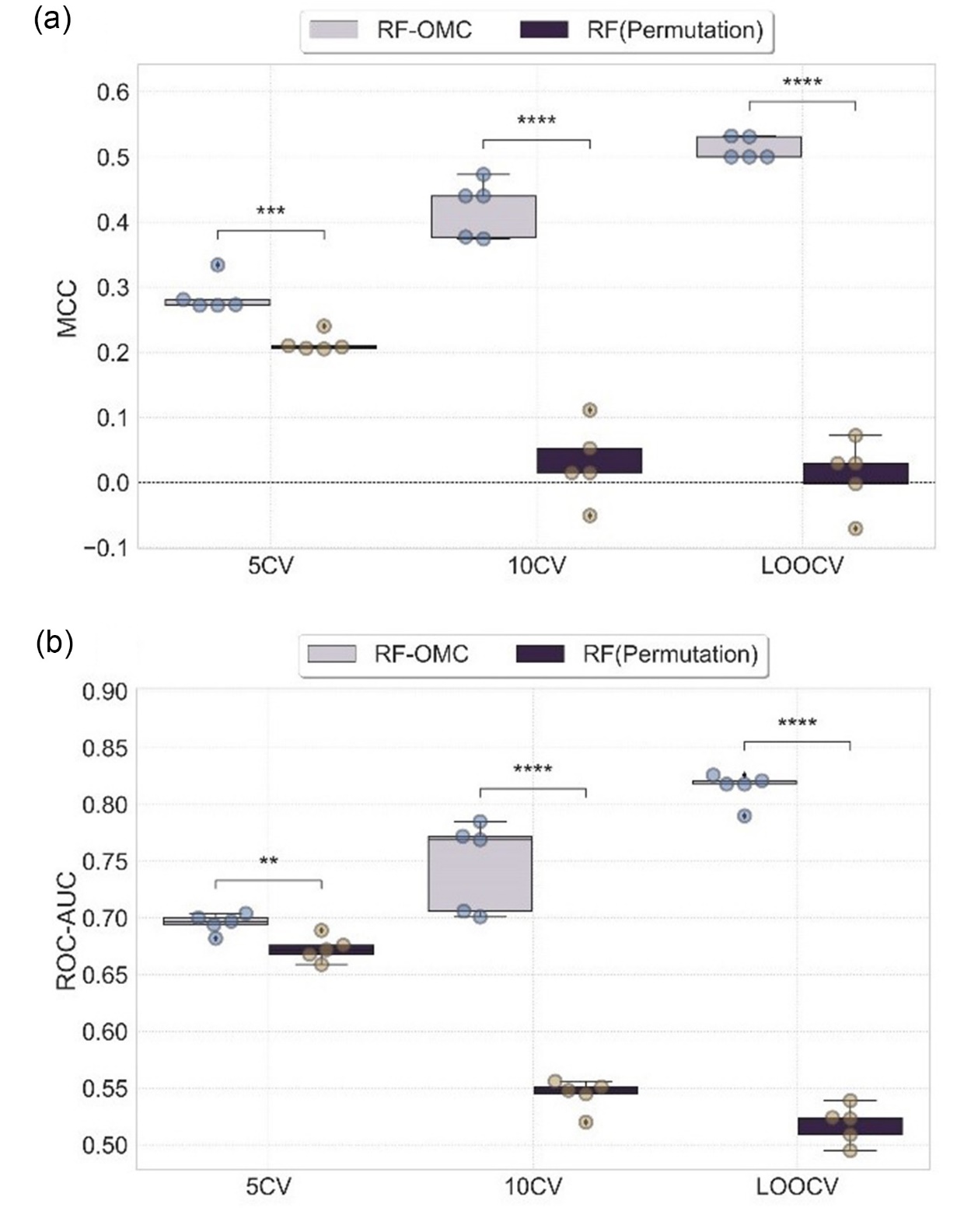


**Figure S6.** **The model performances of the most predictive models compared to those training with class permuted data**. The boxplots present the distributions of MCC (a) and ROC-AUC (b) obtained across five 10-fold CV runs of RF-OMC employed mRNA(FPKM) profile with LOOCV, 5CV and 10CV. RF incorporated OMC feature selection is RF(OMC) and RF trained on the class permutated version of original dataset is RF(Permutation). The model’s predictive performances of OMC and permutation model within each CVs implemented are compared. Each box plot contains the p-value of mean differential expression between 2 groups using a two-sided Welch’s t-test. “****” means p-value ≤ 0.0001. The dashed line represents random classifier at MCC of 0 and ROC-AUC of 0.5. MCC or ROC-AUC of 1 indicate a perfectly accurate prediction. The model performances of OMC model obtain significantly better MCC and ROC-AUC than the permutation model in all the CVs.


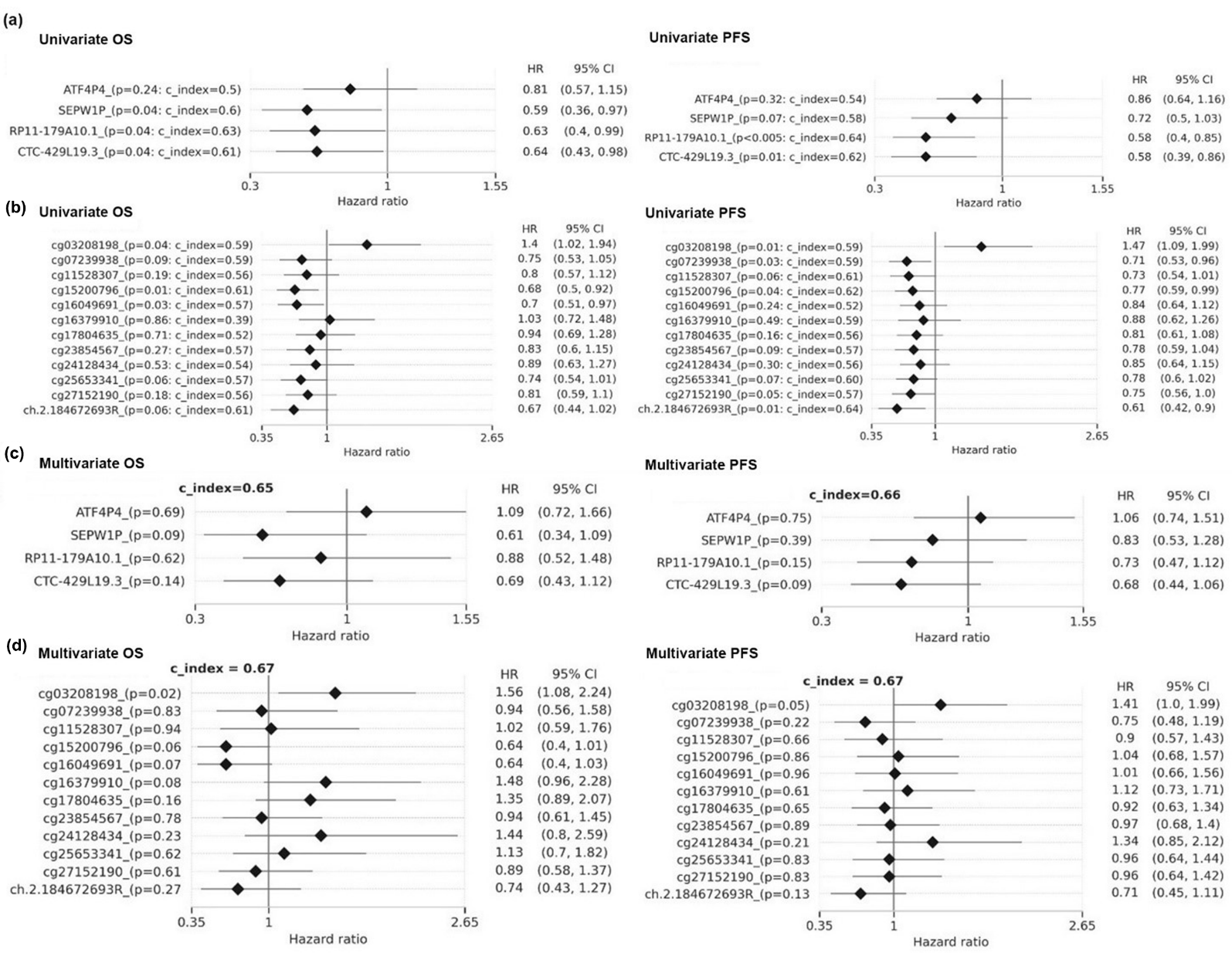


**Figure S7.** **Univariate and multivariate Cox regression analysis of the gemcitabine-response predictors.** The forest plot contains the hazard ratio (HR) and their 95% confidence interval (CI) of univariate (a, b) and multivariate (c, d) Cox regression analysis of 4 predictive genes (a, c) and 12 predictive CpG probes (b, c) related to OS (left) and PFS (right) in PAAD patients treated gemcitabine. A HR greater than 1 indicates that the feature is an independent high-risk factor of death or associated with decrease survival, whereas a HR smaller than 1 indicates this feature is an independent protective factor and associated with improved survival. A hazard ratio of 1 means that there is no difference in survival. The univariate analysis revealed that 3 genes (SEPW1P, RP11-179A10.1, CTC-429L19.3) were significantly (p-value < 0.05) associated with OS and PFS, but they were not significant in multivariate Cox regression analysis. In addition, 3 and 4 CpG probes were significantly (p-value < 0.05) associated with OS and PFS respectively in univariate analysis, but only cg03208198 (HR = 1.56 for OS; HR = 1.41 for PFS) was shown to be significantly (p-value < 0.05) associated with OS and PFS in multivariate analysis. The results suggest that some of the individual gemcitabine-response predictors could be used as independent biomarkers for survival prediction in PAAD gemcitabine-treated patients.


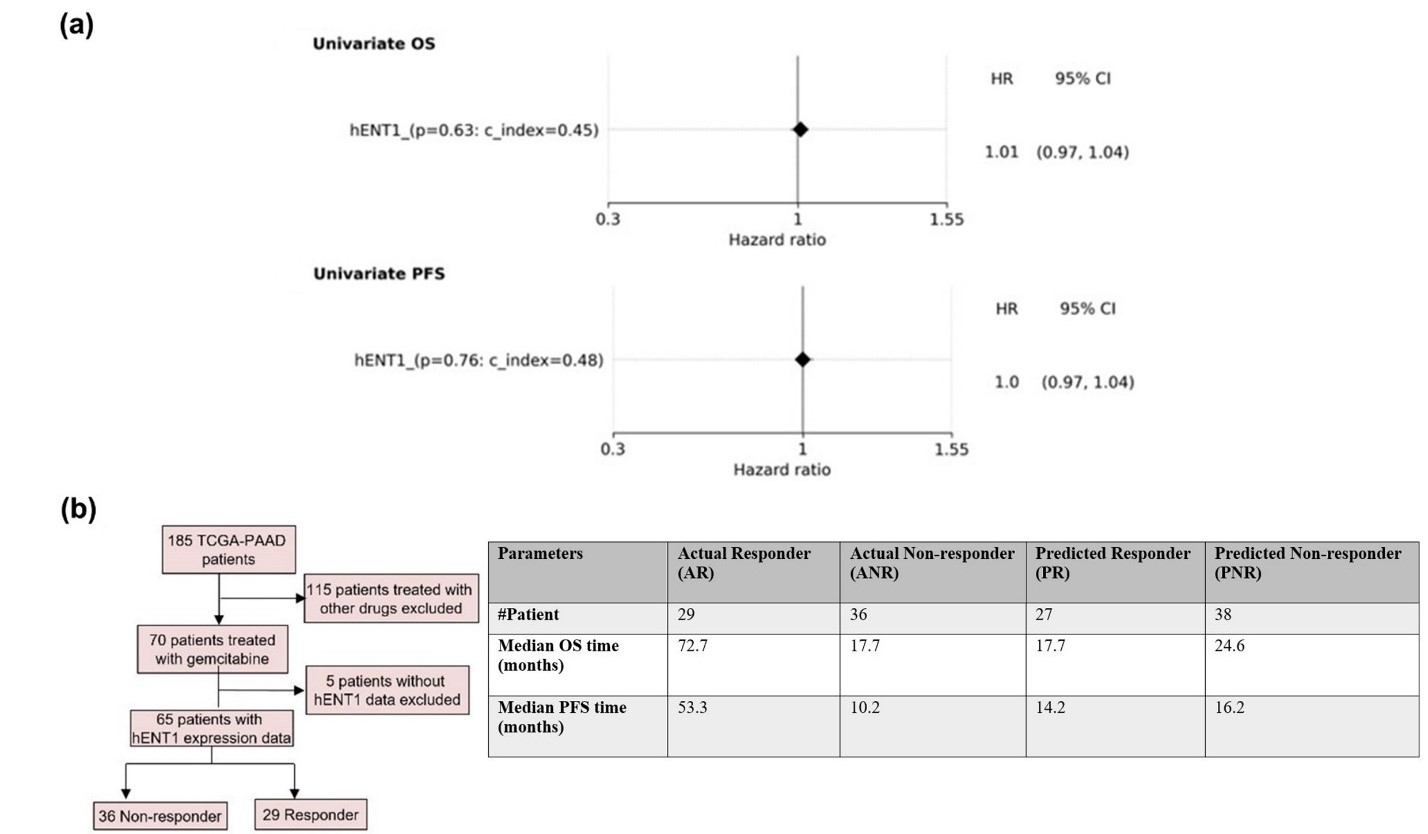


**Figure S8 Univariate Cox regression analysis of hENT1 gene and the comparison between the median OS and PFS time of actual patient response to gemcitabine and those predicted by the hENT1-based RF model.** (a) The forest plot contains the hazard ratio (HR) and their 95% confidence interval (CI) of univariate Cox regression analysis of hENT1 gene related to OS (top) and PFS (bottom) in PAAD patients treated gemcitabine. (b) a flowchart of patient selection and the median OS (defined as the time interval from the date of diagnosis to death or the last known follow-up date) and PFS (defined as the time interval from the date of diagnosis to disease progression or the last known follow-up date) time for four groups, including actual and predicted responders and non-responders by hENT1-based RF model. The median OS and PFS are defined as the time point on the Kaplan-Meier plot where 50% of the patients in each group have survived (for OS) or have not experienced disease progression (for PFS).


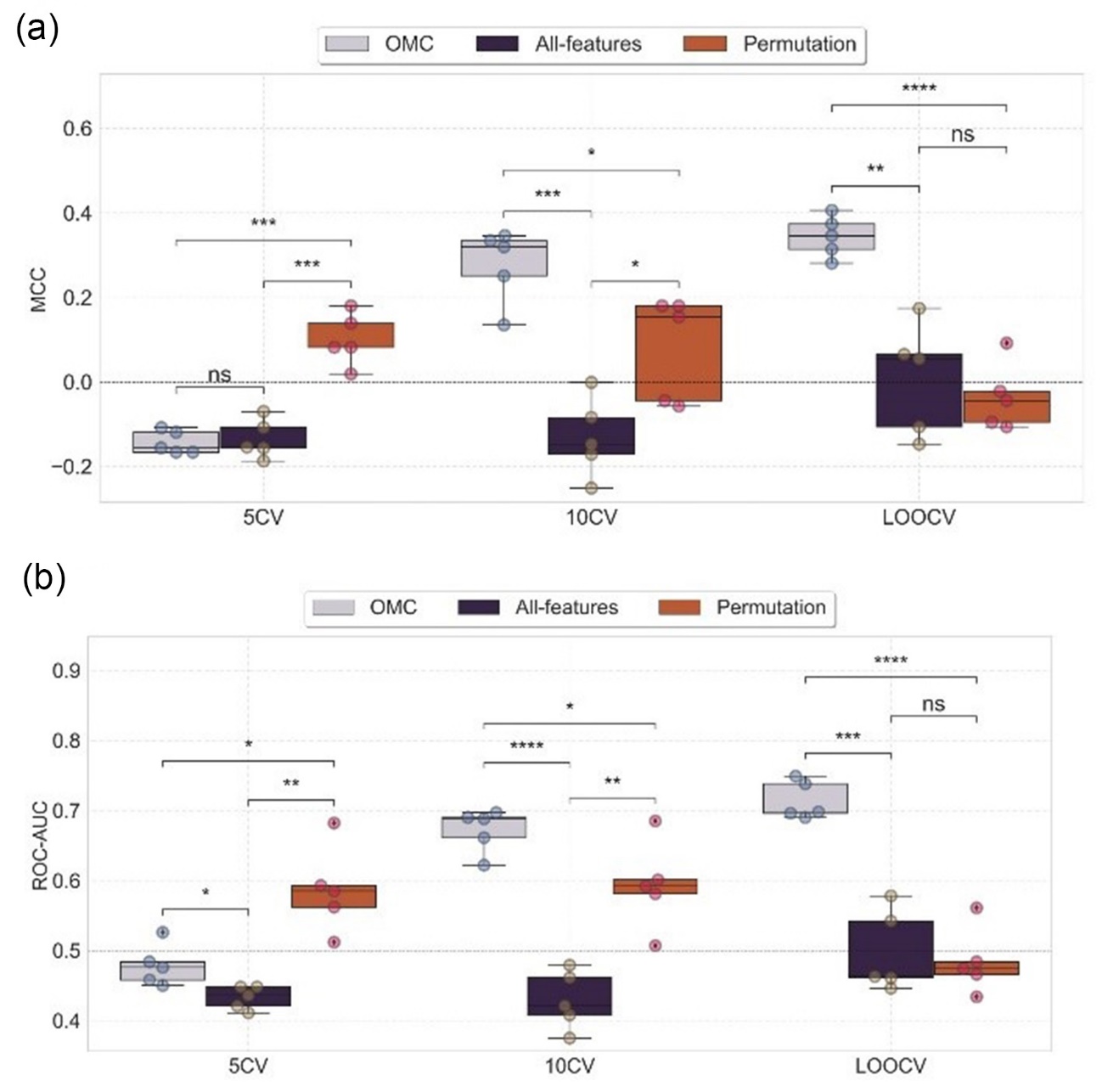


**Figure S9.** **The model performances of the second most predictive models compared to those training with class permuted data**. The boxplots present the distributions of MCC (a) and ROC-AUC (b) obtained across five 10-fold CV runs of XGBoost-OMC employed DNA methylation of CpG profile with LOOCV, 5CV and 10CV. XGBoost incorporated OMC feature selection (XGBoost(OMC)) were represented in green, XGBoost trained on all available 450,000 CpG probes (XGBoost(all-features)) were represented in orange, and XGBoost trained on the class permutated version of original dataset (XGBoost(Permutation)) were represented in purple. The model’s predictive performances within each CVs implemented (OMC vs all-feature and OMC vs permutation) are compared. Each box plot contains the p-value of mean differential expression between 2 groups with a two-sided Welch’s t-test. “****” means p-value ≤ 0.0001. The dashed line represents random classifier at MCC of 0 and ROC-AUC of 0.5. MCC or ROC-AUC of 1 indicate a perfectly accurate prediction.


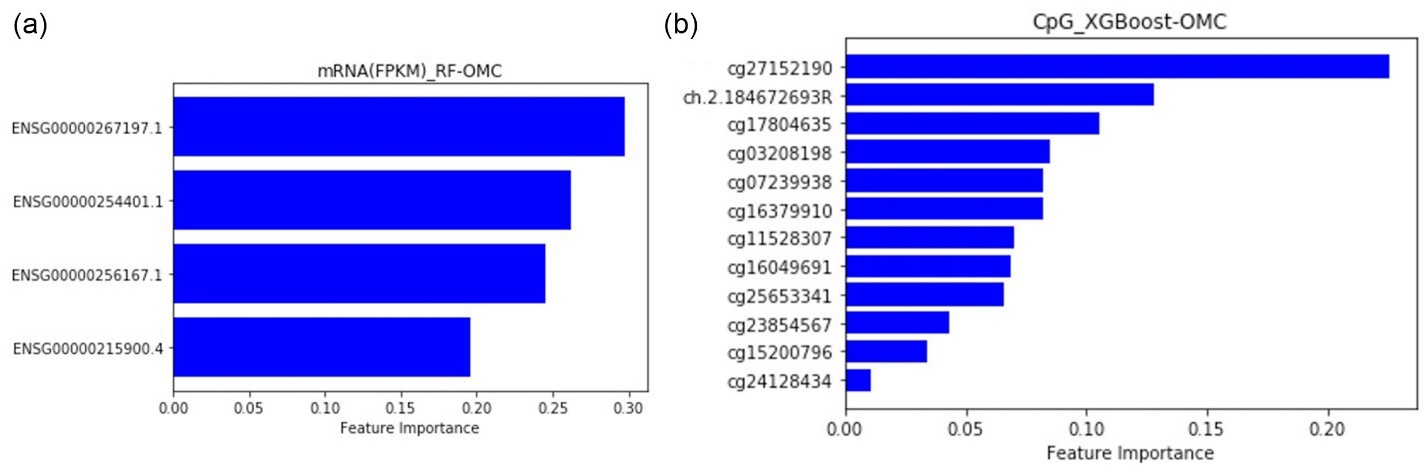


**Figure S10**. **Feature importance scores of the predictive features obtained from the 2 most predictive models for prediction of gemcitabine responses in PAAD patients.** (a) corresponds to RF-OMC incorporating 4 predictive genes, while (b) corresponds to XGBoost-OMC incorporating 12 predictive CpG probes. The reduction in the important scores means less confidence in feature selection for predicting gemcitabine responses in PAAD patients.

Supplementary tables

**Table S1 Clinicopathological characteristics of patients.** All these features were included in building the clinical model, except for vital status and survival data. In order to preprocess the clinical data, each unique label of ordinal features, such as tumor stage, tumor grade, tobacco smoking history, and alcohol history was mapped to an integer value. For instance, the tumor grade contains 3 unique values (grade 1, 2, 3), which were encoded as 1, 2 and 3, respectively. While features with binary values (e.g., gender) were encoded as 0 and 1. The feature with continuous values were binarised into two features according to a given threshold. For example, age was processed using the average age as the threshold, with values below the average being encoded as 0 and the values above the average being encoded as 1. Lastly, the nominal features (e.g., race) were encoded as one-hot encoding.

| Characteristic | | Total | | Responder | | Non-responder | |
| --- | --- | --- | --- | --- | --- | --- | --- |
|  |  | n | % | n | % | n | % |
| Gemcitabine responses | Complete response (CR)  Partial response (PR)  Stable disease (SD)  Progressive disease (PD) | 70 | 100 | 29 | 40.4 | 41 | 58.6 |
|  |  |  |  | CR:24, PR:5 |  | SD: 7, PD:34 |  |
| Gender | Female | 32 | 45.7 | 11 | 37.9 | 21 | 51.2 |
|  | Male | 38 | 54.3 | 18 | 62.1 | 20 | 49.8 |
| Age (years) | Range | 40 - 85 | | 40 - 85 | | 41 - 85 | |
|  | Median ± SD | 66 ± 10.9 | | 65 ± 9.5 | | 66 ± 11.7 | |
| Tumor Grade | G1 | 11 | 15.7 | 4 | 13.8 | 7 | 17.1 |
|  | G2 | 42 | 60.0 | 20 | 69.0 | 22 | 53.7 |
|  | G3 | 17 | 24.3 | 5 | 17.2 | 12 | 29.3 |
| Race | White | 63 | 90.0 | 27 | 93.1 | 36 | 87.8 |
|  | Black or African American | 3 | 4.3 | 1 | 3.4 | 2 | 4.9 |
|  | Asian | 3 | 4.3 | 1 | 3.4 | 2 | 4.9 |
|  | Not reported | 1 | 1.4 | 0 | 0 | 1 | 2.4 |
| Vital Status | Alive | 30 | 42.9 | 20 | 69.0 | 10 | 24.4 |
|  | Dead | 40 | 57.1 | 9 | 31.0 | 31 | 75.6 |
| Histological type | Pancreas-Adenocarcinoma Ductal Type | 66 | 94.3 | 26 | 39.4 | 40 | 60.6 |
|  | Pancreas-Adenocarcinoma Others (NOS) | 4 | 5.7 | 3 | 75 | 1 | 25 |
| Residual Tumor | R0 | 46 | 65.7 | 23 | 50 | 23 | 25 |
|  | R1 | 18 | 25.7 | 4 | 22.2 | 14 | 77.8 |
|  | R2 | 3 | 4.3 | 0 | 0 | 3 | 100 |
|  | Not-reported | 3 | 4.3 | 2 | 66.7 | 1 | 33.3 |
| AJCC  Pathologic T | T1 | 4 | 5.7 | 2 | 50 | 2 | 50 |
|  | T2 | 5 | 7.2 | 1 | 20 | 4 | 80 |
|  | T3 | 56 | 80 | 24 | 42.9 | 32 | 57.1 |
|  | T4 | 4 | 5.7 | 1 | 25 | 3 | 75 |
|  | Not-reported | 1 | 1.4 | 1 | 100 | 0 | 0 |
| AJCC  Pathologic N | N0 | 19 | 27.1 | 10 | 52.6 | 9 | 47.4 |
|  | N1 | 51 | 72.9 | 19 | 37.3 | 32 | 62.7 |
| AJCC  Pathologic M | MX | 37 | 52.9 | 17 | 45.9 | 20 | 54.1 |
|  | M0 | 29 | 41.4 | 12 | 41.4 | 17 | 58.6 |
|  | M1 | 4 | 5.7 | 0 | 0 | 4 | 100 |
| AJCC  Tumor Stage | Stage I & IA | 4 | 5.7 | 2 | 6.9 | 2 | 4.9 |
|  | Stage II (A & B) | 58 | 82.9 | 25 | 86.2 | 33 | 80.5 |
|  | Stage III | 3 | 4.3 | 1 | 3.4 | 2 | 4.9 |
|  | Stage IV | 4 | 5.7 | 0 | 0 | 4 | 9.8 |
|  | Discrepancy | 1 | 1.4 | 1 | 3.4 | 0 | 0 |
| Lymph node count | Median | 15±7.9 |  | 17± 8.3 |  | 13± 7.6 |  |
|  | Range | 1-44 |  | 7-44 |  | 1-35 |  |
| Lymph Node positive by he | Median | 3±2.9 |  | 1±2.5 |  | 3±3.3 |  |
|  | Range | 0-14 |  | 0-11 |  | 0-14 |  |
| Maximum tumor dimension | Median | 3.35±1.4 |  | 3.35±1.0 |  | 3.35±1.9 |  |
|  | Range | 1.8-12 |  | 2-6 |  | 1.8-12 |  |
| Tobacco smoking history | Yes | 58 | 82.9 | 22 | 37.9 | 36 | 62.1 |
|  | Unknown | 12 | 17.1 | 7 | 58.3 | 5 | 41.7 |
| Alcohol history | Yes | 37 | 52.9 | 13 | 35.1 | 24 | 64.9 |
|  | No | 25 | 35.7 | 12 | 48 | 13 | 52 |
|  | Not reported | 8 | 11.4 | 4 | 50 | 4 | 50 |
| History of diabetes | Yes | 16 | 22.9 | 9 | 56.3 | 7 | 43.8 |
|  | No | 40 | 57.1 | 13 | 32.5 | 27 | 67.5 |
|  | Not reported | 14 | 20 | 7 | 50 | 7 | 50 |
| History of chronic pancreatitis | Yes | 3 | 4.3 | 0 | 0 | 3 | 100 |
|  | No | 48 | 68.6 | 18 | 37.5 | 30 | 62.5 |
|  | Not reported | 19 | 27.1 | 11 | 57.9 | 8 | 42.1 |
| Family history of cancer | Yes | 30 | 42.9 | 13 | 43.3 | 17 | 56.7 |
|  | No | 19 | 27.1 | 6 | 31.6 | 13 | 68.4 |
|  | Not reported | 21 | 30 | 10 | 47.6 | 11 | 52.3 |
| Survival (Months) | Overall | 21.1±13.8 |  | 24.9±17.2 |  | 18.4±10.2 |  |
|  | Progression Free | 16.2±12.9 |  | 22.2±16.4 |  | 12.0±7.5 |  |
| *AJCC: The American Joint Committee on Cancer, NOS: Carcinoma not otherwise specified* | | | | | | | |

**Table S2 (related to Figure 4). Expression pattern of the predictive genes and DNA methylation of CpG probes in PAAD patients who respond to gemcitabine**

| **Molecular Features** | **Upregulated** | **Downregulated** | **Total** |
| --- | --- | --- | --- |
| **Genes** | SEPW1P, RP11-179A10.1, ATF4P4, CTC-429L19.3 | None | 4 |
| **Probes** | cg07239938, cg11528307, cg15200796, cg16049691, cg16379910, cg17804635, cg23854567, cg24128434, cg25653341, cg27152190, ch.2.184672693R | cg03208198 | 12 |

**Table S3: Summary of the most predictive models with median MCC (mMCC) at least 0.3**

| Molecular Profile | Algorithm | Median MCC | #All-  features | #Selected features | Features Names | Confusion matrix | | | |
| --- | --- | --- | --- | --- | --- | --- | --- | --- | --- |
|  |  |  |  |  |  | N | | P | |
|  |  |  |  |  |  | TN | FP | FN | TP |
| mRNA (FPKM) | RF-OMC | 0.44 | 60,483 | 4 | SEPW1P, RP11-179A10.1, ATF4P4, CTC-429L19.3 | 27 | 9 | 8 | 21 |
|  | CART-OMC | 0.35 |  | 4 | SEPW1P, RP11-179A10.1, ATF4P4, CTC-429L19.3 | 26 | 10 | 9 | 20 |
|  | KNN-OMC | 0.34 |  | 5 | SEPW1P, RP11-179A10.1, ATF4P4, SPCS2P1, CTC-429L19.3 | 29 | 7 | 14 | 15 |
| mRNA (FPKM-UQ) | LR-OMC | 0.40 | 60,483 | 5 | SEPW1P, RP11-179A10.1, ATF4P4, SPCS2P1, CTC-429L19.3 | 29 | 7 | 12 | 17 |
|  | RF-OMC | 0.31 |  | 7 | SEPW1P, RP11-569A11.1, AC139452.2, RP11-179A10.1, ATF4P4, SPCS2P1, CTC-429L19.3 | 26 | 10 | 11 | 18 |
|  | CART-OMC | 0.30 |  | 6 | SEPW1P, AC139452.2, RP11-179A10.1, ATF4P4, SPCS2P1, CTC-429L19.3 | 26 | 10 | 9 | 20 |
| CpG | XGB-OMC | 0.32 | 450,000 | 12 | cg03208198 (*COL18A1*), cg07239938 (ELA2), cg11528307 (C14orf80 & CRIP1), cg15200796 (TMEM 191C), cg16049691 (AHRR), cg16379910 (B2M), cg17804635 (ZNF703), cg23854567 (PXN), cg24128434 (DNAH2 ), cg25653341 (PLOD3), cg27152190 (HEATR3), ch.2.184672693R. | 31 | 10 | 12 | 17 |

The non-responder was considered as the negative class (N), while the responder was considered as the positive class (P). TP: true positive, TN: true negative, FP: false positive, FN: false negative

**Table: S4 (related to Figure S5). GO enrichment analysis of the genes corresponding to the 12 predictive CpG probes in predicting gemcitabine responses in PAAD patients (Fisher’s exact test corrected with Benjamini-hochberg).**

| GO | Term | Description | Count | Gene | p_value | Fold enrichment | Correct p-value |
| --- | --- | --- | --- | --- | --- | --- | --- |
| BP | GO:0042221 | Response to chemical | 8 | COL18A1, PXN, ZNF703, AHRR, PLOD3, CRIP1, B2M, ELANE | 0.0005 | 3.443 | 0.39 |
|  | GO:0007155 | Cell adhesion | 5 | COL18A1, PXN, ZNF703, B2M, ELANE | 0.0022 | 6.921 | 0.43 |
|  | GO:0022610 | Biological adhesion | 5 | COL18A1, PXN, ZNF703, B2M, ELANE | 0.0023 | 6.891 | 0.43 |
|  | GO:0098602 | Single organism cell adhesion | 4 | PXN, ZNF703, B2M, ELANE | 0.0025 | 11.89 | 0.43 |
|  | GO:0071822 | Protein complex subunit organization | 5 | COL18A1, DNAH2, PXN, PLOD3, B2M | 0.0026 | 6.615 | 0.43 |
|  | GO:1901700 | Response to oxygen-containing compound | 5 | COL18A1, PXN, ZNF703, B2M, ELANE | 0.0039 | 5.955 | 0.53 |
|  | GO:0097435 | Supramolecular fiber organization | 4 | COL18A1, PXN, PLOD3, B2M | 0.0047 | 9.534 | 0.55 |
|  | GO:0001886 | Endothelial cell morphogenesis | 2 | COL18A1, PLOD3 | 0.0054 | 330.7 | 0.55 |
|  | GO:0070887 | Cellular response to chemical stimulus | 6 | PXN, ZNF703, AHRR, PLOD3, CRIP1, B2M | 0.0064 | 3.721 | 0.58 |
|  | GO:0010033 | Response to organic substance | 6 | PXN, ZNF703, PLOD3, CRIP1, B2M, ELANE | 0.0073 | 3.611 | 0.6 |
|  | GO:0043933 | Macromolecular complex subunit organization | 5 | COL18A1, DNAH2, PXN, PLOD3, B2M | 0.010 | 4.568 | 0.76 |
|  | GO:0030198 | Extracellular matrix organization | 3 | COL18A1, PLOD3, ELANE | 0.012 | 15.14 | 0.79 |
|  | GO:0043062 | Extracellular structure organization | 3 | COL18A1, PLOD3, ELANE | 0.012 | 15.10 | 0.79 |
|  | GO:0003382 | Epithelial cell morphogenesis | 2 | COL18A1, PLOD3 | 0.014 | 122.9 | 0.84 |
|  | GO:0022407 | Regulation of cell-cell adhesion | 3 | ZNF703, B2M, ELANE | 0.017 | 12.75 | 0.92 |
|  | GO:0071495 | Cellular response to endogenous stimulus | 4 | PXN, ZNF703, PLOD3, B2M | 0.018 | 5.894 | 0.92 |
|  | GO:0010035 | Response to inorganic substance | 3 | COL18A1, CRIP1, B2M | 0.024 | 10.678 | 0.99 |
|  | GO:0016337 | Single organismal cell-cell adhesion | 3 | ZNF703, B2M, ELANE | 0.027 | 10.02 | 0.99 |
|  | GO:0019731 | Antibacterial humoral response | 2 | B2M, ELANE | 0.029 | 61.43 | 0.99 |
|  | GO:0001885 | Endothelial cell development | 2 | COL18A1, PLOD3 | 0.030 | 58.90 | 0.99 |
|  | GO:0044707 | Single-multicellular organism process | 7 | COL18A1, PXN, ZNF703, PLOD3, CRIP1, B2M, ELANE | 0.030 | 2.150 | 0.99 |
|  | GO:0042127 | Regulation of cell proliferation | 4 | COL18A1, ZNF703, B2M, ELANE | 0.033 | 4.72 | 0.99 |
|  | GO:0022607 | Cellular component assembly | 5 | DNAH2, PXN, ZNF703, PLOD3, B2M | 0.033 | 3.268 | 0.99 |
|  | GO:0043623 | Cellular protein complex assembly | 3 | DNAH2, PXN, B2M | 0.033 | 9.008 | 0.99 |
|  | GO:0016043 | Cellular component organization | 7 | COL18A1, DNAH2, PXN, ZNF703, PLOD3, B2M, ELANE | 0.034 | 2.094 | 0.99 |
|  | GO:0009719 | Response to endogenous stimulus | 4 | PXN, ZNF703, PLOD3, B2M | 0.034 | 4.616 | 0.99 |
|  | GO:0051674 | Localization of cell | 4 | DNAH2, PXN, ZNF703, ELANE | 0.036 | 4.528 | 0.99 |
|  | GO:0048870 | Cell motility | 4 | DNAH2, PXN, ZNF703, ELANE | 0.036 | 4.528 | 0.99 |
|  | GO:0000904 | Cell morphogenesis involved in differentiation | 3 | COL18A1, PXN, PLOD3 | 0.039 | 8.206 | 0.99 |
|  | GO:0050829 | Defence response to Gram-negative bacterium | 2 | B2M, ELANE | 0.039 | 44.33 | 0.99 |
|  | GO:0050896 | Response to stimulus | 8 | COL18A1, PXN, ZNF703, AHRR, PLOD3, CRIP1, B2M, ELANE | 0.040 | 1.744 | 0.99 |
|  | GO:0071840 | Cellular component organization or biogenesis | 7 | COL18A1, DNAH2, PXN, ZNF703, PLOD3, B2M, ELANE | 0.040 | 2.031 | 0.99 |
|  | GO:0030155 | Regulation of cell adhesion | 3 | ZNF703, B2M, ELANE | 0.042 | 7.803 | 0.99 |
|  | GO:0008283 | Cell proliferation | 4 | COL18A1, ZNF703, B2M, ELANE | 0.043 | 4.242 | 0.99 |
|  | GO:0030855 | Epithelial cell differentiation | 3 | COL18A1, ZNF703, PLOD3 | 0.043 | 7.808 | 0.99 |
|  | GO:0044085 | Cellular component biogenesis | 5 | DNAH2, PXN, ZNF703, PLOD3, B2M | 0.044 | 2.986 | 0.99 |
|  | GO:0030199 | Collagen fibril organization | 2 | COL18A1, PLOD3 | 0.045 | 30.09 | 0.99 |
|  | GO:0061844 | Antimicrobial humoral immune response mediated by antimicrobial peptide | 2 | B2M, ELANE | 0.048 | 36.43 | 1 |
|  | GO:0045446 | Endothelial cell differentiation | 2 | COL18A1, PLOD3 | 0.049 | 35.54 | 1 |
| CC | GO:0005788 | Endoplasmic reticulum lumen | 3 | COL18A1, PLOD3, B2M | 0.008 | 19.20 | 1 |
|  | GO:0032991 | Macromolecular complex | 7 | COL18A1, DNAH2, PXN, ZNF703, AHRR, B2M, ELANE | 0.026 | 2.321 | 1 |
|  | GO:0035580 | Specific granule lumen | 2 | B2M, ELANE | 0.027 | 65.05 | 1 |
| MF | GO:0043167 | Ion binding | 7 | COL18A1, DNAH2, PXN, ZNF703, PLOD3, CRIP1, ELANE | 0.024 | 2.237 | 1 |
|  | GO:0001191 | Transcriptional repressor activity, RNA polymerase II transcription factor binding | 2 | AHRR, ELANE | 0.044 | 39.535 | 1 |
| BP: Biological Process, CC: Cellular Component, MF: Molecular Function | | | | | |  |  |

**Table S5.** **KEGG pathway enrichment analysis of the genes associated with the 12 predictive CpG probes in predicting PAAD treated gemcitabine (Fisher’s exact test corrected with Benjamini-hochberg).**

| Pathway | ID | Gene | p-value | Enrichment ratio | Corrected p-value |
| --- | --- | --- | --- | --- | --- |
| Other types of O-glycan biosynthesis | hsa00514 | PLOD3 | 0.007 | 0.0454 | 0.052 |
| Tryptophan metabolism | hsa00380 | AHRR | 0.013 | 0.0238 | 0.052 |
| Ovarian steroidogenesis | hsa04913 | AHRR | 0.015 | 0.0204 | 0.052 |
| Lysine degradation | hsa00310 | PLOD3 | 0.018 | 0.0169 | 0.052 |
| Steroid hormone biosynthesis | hsa00140 | AHRR | 0.018 | 0.0166 | 0.052 |
| Retinol metabolism | hsa00830 | AHRR | 0.021 | 0.0149 | 0.052 |
| Metabolism of xenobiotics by cytochrome P450 | hsa00980 | AHRR | 0.023 | 0.0131 | 0.052 |
| Antigen processing and presentation | hsa04612 | B2M | 0.024 | 0.0130 | 0.052 |
| Chemical carcinogenesis | hsa05204 | AHRR | 0.025 | 0.0121 | 0.052 |
| Protein digestion and absorption | hsa04974 | COL18A1 | 0.027 | 0.0111 | 0.052 |
| Systemic lupus erythematosus | hsa05322 | ELANE | 0.040 | 0.0075 | 0.069 |

**Table S6 Comparison of predictive performances of five 10-fold CV runs of the 2 most predictive models: RF-OMC employed 4 predictive mRNAs (mRNA(FPKM)_RF-OMC) and XGBoost employed 12 predictive DNA methylation of CpG probes (CpG_XGBoost-OMC) to those using hENT1 gene expression only (hENT1-RF, and hENT1-XGBoost) in predicting gemcitabine responses of PAAD patients**

| Random seed | MCC | | | | ROC-AUC | | | |
| --- | --- | --- | --- | --- | --- | --- | --- | --- |
|  | hENT1-RF | hENT1-XGBoost | mRNA(FPKM)_RF-OMC | CpG-XGBoost-OMC | hENT1-RF | hENT1-XGBoost | mRNA(FPKM)_RF-OMC | CpG_XGBoost-OMC |
| 1 | -0.066 | -0.045 | 0.44 | 0.334 | 0.47 | 0.468 | 0.785 | 0.688 |
| 2 | -0.066 | -0.045 | 0.473 | 0.251 | 0.469 | 0.464 | 0.772 | 0.661 |
| 3 | -0.066 | -0.045 | 0.44 | 0.32 | 0.475 | 0.467 | 0.769 | 0.697 |
| 4 | -0.066 | -0.045 | 0.374 | 0.135 | 0.4768 | 0.48 | 0.701 | 0.622 |
| 5 | -0.066 | -0.045 | 0.377 | 0.346 | 0.469 | 0.472 | 0.706 | 0.69 |

**Table S7 The 4 predictive mRNA features obtained from RF-OMC built on mRNA profile achieving the highest mMCC of 0.44**

| Gene_Ensemble_ID | Gene_Symbol | Gene_Type |
| --- | --- | --- |
| ENSG00000254401.1 | RP11-179A10.1 | RNA Gene |
| ENSG00000267197.1 | CTC-429L19.3 | RNA Gene |
| ENSG00000256167.1 | ATF4P4 | Pseudogene |
| ENSG00000215900.4 | SEPW1P | Pseudogene |

**Table S8 CpG probes selected by the OMC algorithms using thte DNA methylation of CpG profile achieving the median MCC (mMCC) of 0.1**

| XGBoost-OMC  selected Probe |  | LGBM-OMC  selected Probe | CART-OMC  selected Probe |  | | RF-OMC  selected Probe | Gene  Symbol | Gene Type | Feature  Type |
| --- | --- | --- | --- | --- | --- | --- | --- | --- | --- |
| mMCC = 0.32 |  | mMCC = 0.18 | mMCC = 0.28 |  | | mMCC = 0.18 |  |  |  |
| cg11528307 | | cg11528307 | cg11528307 | | cg11528307 | | C14orf80 | Protein coding | Island |
|  |  |  |  |  |  | | CRIP1 | Protein coding |  |
| ch.2.184672693R | | ch.2.184672693R | ch.2.184672693R | | ch.2.184672693R | | - | - | - |
| cg17804635 | | cg17804635 |  |  | |  | ZNF703 | Protein coding | Island |
| cg03208198 | | cg03208198 |  |  | |  | COL18A1 | Protein coding | Island |
|  |  |  |  |  | |  | MIR6815 | miRNA | Island |
| cg07239938 | | cg07239938 |  |  | |  | ELANE | Protein coding | N_Shore |
| cg15200796 | | cg15200796 |  |  | |  | TMEM191C | lincRNA | Island |
| cg16049691 | | cg16049691 |  |  | |  | AHRR | Protein coding | - |
| cg16379910 | | cg16379910 |  |  | |  | B2M |  |  |
| cg23854567 | | cg23854567 |  |  | |  | PXN | Protein coding | - |
| cg24128434 | | cg24128434 |  |  | |  | DNAH2 | Protein coding | S_Shelf |
| cg25653341 | | cg25653341 |  |  | |  | PLOD3 | Protein coding | S_Shelf |
| cg27152190 | | cg27152190 |  |  | |  | RP11-429P3.3 | antisense | N_Shelf |
